# Modelling amorphous materials *via* a joint solid-state NMR and X-ray absorption spectroscopy and DFT approach: application to alumina†

**DOI:** 10.1039/d2sc04035b

**Published:** 2022-12-21

**Authors:** Angela F. Harper, Steffen P. Emge, Pieter C. M. M. Magusin, Clare P. Grey, Andrew J. Morris

**Affiliations:** a Theory of Condensed Matter, Cavendish Laboratory, University of Cambridge J. J. Thomson Avenue Cambridge CB3 0HE UK; b Yusuf Hamied Department of Chemistry, University of Cambridge Lensfield Road Cambridge CB2 1EW UK; c Institute for Life Sciences & Chemistry, Hogeschool Utrecht Heidelberglaan 7 3584 CS Utrecht Netherlands; d School of Metallurgy and Materials, University of Birmingham Edgbaston Birmingham B15 2TT UK a.j.morris.1@bham.ac.uk

## Abstract

Understanding a material's electronic structure is crucial to the development of many functional devices from semiconductors to solar cells and Li-ion batteries. A material's properties, including electronic structure, are dependent on the arrangement of its atoms. However, structure determination (the process of uncovering the atomic arrangement), is impeded, both experimentally and computationally, by disorder. The lack of a verifiable atomic model presents a huge challenge when designing functional amorphous materials. Such materials may be characterised through their local atomic environments using, for example, solid-state NMR and XAS. By using these two spectroscopy methods to inform the sampling of configurations from *ab initio* molecular dynamics we devise and validate an amorphous model, choosing amorphous alumina to illustrate the approach due to its wide range of technological uses. Our model predicts two distinct geometric environments of AlO_5_ coordination polyhedra and determines the origin of the pre-edge features in the Al K-edge XAS. From our model we construct an average electronic density of states for amorphous alumina, and identify localized states at the conduction band minimum (CBM). We show that the presence of a pre-edge peak in the XAS is a result of transitions from the Al 1s to Al 3s states at the CBM. Deconvoluting this XAS by coordination geometry reveals contributions from both AlO_4_ and AlO_5_ geometries at the CBM give rise to the pre-edge, which provides insight into the role of AlO_5_ in the electronic structure of alumina. This work represents an important advance within the field of solid-state amorphous modelling, providing a method for developing amorphous models through the comparison of experimental and computationally derived spectra, which may then be used to determine the electronic structure of amorphous materials.

## Abstract 2

A first principles model of amorphous Al_2_O_3_ is constructed which reproduces the features of experimental ^27^Al NMR and Al K-edge XAS spectra on an ALD-deposited sample of amorphous Al_2_O_3_.

## Graphical abstract

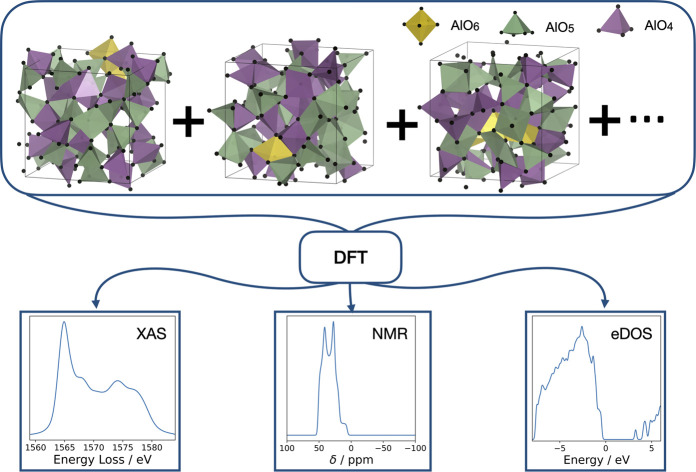

## Introduction

Atomic level quantum mechanical modelling has played a critical role in driving many of the advances made involving crystalline materials over the last two decades – first in terms of rationalizing materials properties, but now increasingly in predicting and optimizing both materials and devices.^1–4^ A significant challenge, however, lies in applying the same quantum mechanical methods to amorphous materials,^4,5^ despite their increasing role in devices including dielectric layers in organic electronics, and as protective coatings across a range of materials.^6–10^

Crystal structures, by definition, contain translational and point-group symmetries. The exploitation of these symmetries reduces the number of degrees of freedom needed to fully describe the arrangement atoms in the system, consequently making them easier to model. Amorphous structures lack the symmetries present in crystalline materials, and therefore require large simulation cells to capture the disorder of their constituent atoms, leading to very expensive or sometimes prohibitively large calculations.

To understand and ultimately improve the electronic properties of an amorphous material, it is imperative to produce an accurate, verifiable, model of its local atomic and electronic structure. In addition to the computational constraints, it is also non-trivial to validate such a model against relevant experimental structural data. Most structural determination of amorphous solids uses structure factors and radial distribution functions,^11–14^ especially on well-studied systems such as amorphous carbon, silicon, and silica. However, these provide only coarse-grained structure property relationships across the average structure such as bond lengths, average coordination number, and bond angles. The complete structure solution of amorphous materials ultimately relies on locally sensitive, element specific techniques such as Nuclear Magnetic Resonance (NMR) and X-ray Absorption Spectroscopy (XAS). The advantage of these techniques is that they are element specific and allow for angstrom-level resolution of the atomic structure, and in the case of XAS enable the understanding of oxidation states and electronic structure.

Recent progress in machine learning has enabled models of amorphous single element structures such as silicon, carbon, and phosphorus,^4,15–17^ as well as progress in some two-element systems including HfO_2_ and Li_*x*_Si.^3,18^ In these methods, machine learning is applied to describe the atomic level interactions using training sets from density-functional theory (DFT) energy and force calculations; machine-learned models therefore have comparable radial distribution factors, bond-lengths, and structure factors to DFT-derived models with improved simulation time and length scales.^3^ However, their ability to predict spectral properties such as NMR and XAS with first-principles accuracy is lacking; although ShiftML predicts NMR shifts for molecular solids, its nuclei are limited to ^13^C, ^1^H, ^15^N, ^17^O, and ^33^S.^19^ Similarly, XAS spectral lines for transition-metal oxides are predicted using a random-forest method but applied only to a set of known, crystalline, transition-metal oxides.^20^ Clearly, there is a need for spectral predictions with first-principles accuracy, which can be applied to amorphous materials in general.

The phase-space of crystalline and amorphous alumina (am-Al_2_O_3_) is vast, with at least eight known crystalline phases, which are detailed in a series of reviews over the last two decades.^21–23^ All of the crystalline phases are characterized by the stacking of either four-fold coordinated tetrahedral Al(iv) or six-fold coordinated octahedral Al(vi) subunits. Only the amorphous phase of alumina contains five-fold coordinated Al(v) subunits, thereby distinguishing it from the crystalline phases. Experimental ^27^Al NMR shows that the ratios of Al(vi : v : vi) environments present in amorphous alumina varies widely with synthesis technique, deposition temperature, and the substrate onto which alumina is deposited.^24,25^ Despite the large number of experimental studies showing that am-Al_2_O_3_ has several different structures, the computational literature^26–29^ is still in need of an am-Al_2_O_3_ model which is compared directly to such detailed experimental spectroscopy results; at present the only comparison is to the 1997 neutron diffraction experiments by Lamparter and Kniep^30^ on anodic alumina.

Of the various methods for the preparation of alumina, atomic layer deposition (ALD) is one of the most common, because of the ability to deposit single atomic-layers. ALD deposited alumina has well-understood surface chemistry,^31,32^ and was first used as a high-*k* dielectric material, and is now used as a coating layer across a range of electronic devices. Am-Al_2_O_3_ is a wide band-gap insulator which enables surface passivation, interface stability, and protects against degradation as a coating material.^6–8,33,34^ These properties have increased the capacity retention of Li-ion battery electrodes,^33–35^ enhanced the lifetime of perovskite solar cells,^6,36^ and improved the catalytic capabilities of metal–organic frameworks.^37,38^ Given its clear widespread applications, an atomic level model specific to ALD deposited am-Al_2_O_3_ would be indispensable to the device physics community.

This article presents our method for modelling the local structure of an amorphous material, by exploiting both first-principles calculations and high-quality experimental spectroscopy to am-Al_2_O_3_ ’s detailed electronic structure. The method is general and we use am-Al_2_O_3_ as an illustrative example. The novelty of our approach lies in the integration of locally sensitive experimental spectroscopy techniques with spectroscopy calculated from first principles. We obtain NMR spectra at fields up to 1 GHz, which is the current state-of-the-art in solid-state NMR, and show that our DFT-based model contains the same structural features captured by these experimental measurements. The atomic level accuracy of this model enables us to identify two distinct five-fold coordination geometries present in am-Al_2_O_3_, and the orbital character of electronic states at the Al pre-edge of the absorption spectrum, which are unique to am-Al_2_O_3_. We finally calculate an average electronic density of states (eDOS) for our model, thereby opening the door for further investigation into amorphous electronic structure.

## Methods

### Experimental methods

Substrates for ALD were washed with acetone, methanol/ethanol, then deionized water and blow dried with N_2_ before deposition. Depositions were performed with a Picosun R-200 Advanced ALD tool attached to an MBraun glovebox. At a chamber base pressure of approximately 12 hPa the substrates were heated to 150 °C. Trimethylaluminum (TMAl, EpiValence Ltd, Electronic grade) was used as precursor gas, deionized water as reactant and N_2_ as purging gas. For each cycle, corresponding to one layer, the substrates were first exposed to precursor gas (flow = 150 sccm), purged, then reactant gas (flow = 200 sccm) and finally the chamber was purged again which completes a cycle. Pulse and purge durations were 0.1 s and 10 s, respectively.

For synchrotron measurements, Al_2_O_3_ ALD films were deposited on pre-cut 3 mm × 3 mm Si substrates (Pi-KEM, Prime Grade, intrinsic dopant). The substrates originated from a diced 2 inch diameter Si(100) wafer (275 ± 25 μm thick, >200 Ω cm) single-side polished with a thermal dry oxide layer of 20 nm ± 10% on both sides. Substrates with varying numbers of ALD layers were prepared, ranging from 2 to 1000 layers. XAS measurements shown in this work were obtained from the 1000 layer sample. Since more material was required for the NMR measurements, an 8 inch Si wafer (Picosun) was used and 1000 ALD layers were deposited. Al K-edge XAS experiments were conducted at the I09 beamline at Diamond Light Source (United Kingdom) in a total electron yield setup. Data was collected across the 1555–1600 eV range at a step size of 200 meV, using the 1.5 keV X-ray. The experimental data was then referenced against gold 4f foil using the same beamline setup.

The NMR spectroscopy measurements were performed at three different field strengths, 16.4 T, 11.8 T, and 23.5 T as detailed below. 16.4 T & 11.8 T: ^27^Al NMR spectra were acquired using Bruker 4 mm HXY MAS probes. 1D spectra were acquired using a one-pulse pulse program with a small flip angle (π/6) on Bruker 700 MHz (16.4 T) and 500 MHz (11.8 T) magnets with Avance III consoles. Two-dimensional ^27^Al 3QMAS (triple quantum MAS) NMR spectra were acquired on a Bruker 700 MHz (16.4 T) magnet. Quadrupolar pulse optimization was performed on γ-Al_2_O_3_ powder (Acros Organics). 23.5 T: ^27^Al NMR spectra were acquired using a Bruker 1.9 mm HXY MAS probe. Spectra were acquired using a rotor-synchronised (40 kHz ≅ 2*τ* = 50 μs) Hahn-echo spectrum with p2=2·p1 = 5 μs (π/2 = 2.5 μs) on a Bruker 1.0 GHz (23.5 T) magnet with an Avance Neo console. The MAS NMR experiments were performed at sample spinning speeds of 14 kHz (16.4 T & 11.8 T) or 40 kHz (23.5 T). The spectra were externally referenced to AlF_3_ powder (−17 ppm^39^). The NMR sample was obtained by scratching off the top layer deposited on the 8 inch wafer using a Wolfram carbide pen. The powder was packed into a 4 mm (16.4 T & 11.8 T) or 1.9 mm (23.5 T) ZrO_2_ rotor.

Bruker Topspin software was used for raw data handling and processing. The ^27^Al spectra were fitted with DMFIT software^40^ to obtain integrated ratios and values of average isotropic shift (*δ*_iso_), average quadrupolar coupling constant (*C*_Q_) and the chemical shift distribution (ΔCS) using the CzSimple model with *d* = 5 for the Gaussian Isotropic Model (GIM) case. The best fit is obtained using the GIM case of the Czjzek model,^41,42^ corresponding to a distribution of local environments that lead to a spread of quadrupolar coupling constants and chemical shifts.

### DFT-calculated spectroscopy

Details of the methods used to generate the amorphous model are given in the Amorphous model generation section. All *ab initio* molecular dynamics (AIMD) simulations were performed using the VASP v5.4.1 DFT package^43^ in the NVT ensemble using a Nose–Hoover thermostat^44^ and with projector-augmented wave pseudopotentials with a plane-wave energy cutoff of 520 eV,^45^ the PBE functional, and sampling the Brillouin zone at the *Γ* point. The specific pseudopotentials, parameters, and methods used to calculate the spectroscopy from first-principles are described in this section.

Both the XAS and NMR spectra were calculated on each configuration in the model independently and the outputs were summed across configurations to calculate the total spectrum. All spectral calculations, and eDOS were calculated at a plane-wave energy cut-off of 1000 eV and a single *k*-point at *Γ* in CASTEP v19.11.^46^ The CASTEP gauge-inducing projector augmented wave (GIPAW) method was used to calculate all NMR parameters,^46,47^ with a PBE exchange-correlation functional and using CASTEP's on-the-fly generated ultrasoft C18 library of pseudopotentials. The NMR parameters were averaged over 45 configurations from AIMD with 48 Al atoms in each cell totalling 2160 ^27^Al NMR chemical shift parameters for the am-Al_2_O_3_ model.

As ^27^Al is a quadrupolar nuclei (*I* = 5/2), quadrupolar effects play a role in the resulting experimental NMR lineshape. However, the DFT-calculated NMR does not *a priori* include the effects of the quadrupolar nuclei in the calculation of the chemical shift parameters, and therefore we chose to additionally perform spin-simulations on each Al atom in the model, to obtain a DFT-calculated spectra including quadrupolar effects. Using the spin-simulation software, SIMPSON^48^ we carried out spin-simulations on each Al atom in the model, using the same spinning speeds and magnetic fields as in the experimental NMR (4 kHz (16.4 T & 11.8 T) and 40 kHz (23.5 T)). The spin-simulations used *C*_Q_, the isotropic chemical shift (*δ*_iso_), and asymmetry parameter of the electric-field gradient (*η*_Q_) from DFT. These spectra were obtained without including any broadening in the frequency spectrum and were subsequently broadened using a Gaussian broadening scheme to produce the spectra shown in Fig. 4.

To calculate the 2D isotropic *vs.* quadrupolar shift from the DFT-calculated NMR parameters, the quadrupolar induced shift, *δ*_qs_, was calculated as follows,^41^
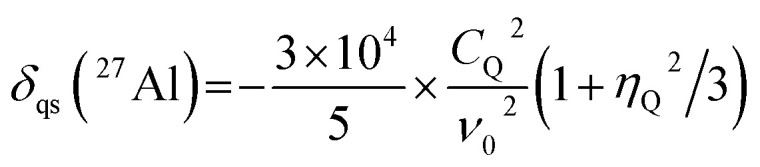
where *ν*_0_ is the Larmor frequency in MHz. Both *C*_Q_ and *η*_Q_ are obtained from the diagonalized EFG tensor calculated using DFT. A spectrometer frequency of 182.4 MHz for ^27^Al, corresponding to a ^1^H Larmor frequency of 700 MHz, was used for *ν*_0_.

The total Al K-edge XAS for am-Al_2_O_3_ was calculated as an average across each of the 45 randomly selected configurations. In each configuration, a single Al atom was chosen as the site at which to calculate the Al K-edge XAS. The Al atoms were chosen by randomly selecting Al atoms across the configurations which satisfied the distribution of the 50%, 38%, and 12% of Al(iv : v : vi) coordination environments present in the model. Each individual XAS spectrum was calculated using the core-hole pseudopotential method within CASTEP v19.11.^49,50^ To calculate the XAS for the Al K-edge, a pseudopotential with a 1s core-hole was placed on the selected Al atom and charged balanced by placing a total positive charge of +1 on the cell. The absorption spectrum was calculated at a plane-wave energy cut-off of 1000 eV, using the “hard” pseudopotential library of ultrasoft pseudopotentials in CASTEP v19.11,^50^ which include an additional set of semicore states. The absorption spectrum was produced by OptaDOS and broadened using the adaptive broadening scheme.^51–53^ This spectrum was referenced using the method of Mizoguchi *et al.*^54^ such that the transition energy is referenced to the difference in energy between the ground-state configuration of am-Al_2_O_3_ and the configuration which includes the core-hole pseudopotential.

## Results

### Amorphous model generation

The ideal amorphous model would comprise a highly accurate quantum mechanical description of interatomic interactions in a very large simulation cell. However, first-principles quantum mechanical calculations using DFT scale as O(N^3^) making these large cell calculations unfeasible. Hence amorphous modellers choose a tradeoff between large supercells which have a high degree of disorder but with atomic interactions described by classical interatomic potentials, or smaller cells at a quantum mechanical level of theory, and a limited description of the structural disorder. Thus, all previous models of amorphous alumina from first principles are on the order of 50 to 200 atoms, and any observables such as the eDOS or chemical shift consider only a single unit cell.^28,55–57^

Experimental ^27^Al NMR on alumina shows that the amorphous structure is very sensitive to synthesis properties including temperature, substrate and deposition type.^24^ However, most models of am-Al_2_O_3_ in the literature^26–29,56^ only validate their model against the radial distribution function (RDF) from the Lamparter *et al.* 1997 study on am-Al_2_O_3_ prepared by anodization.^30^ Given the known interdependence between structure and deposition conditions, especially in the case of amorphous alumina, a direct comparison is imperative, as we will show herein.^58^

**Fig. 1:**
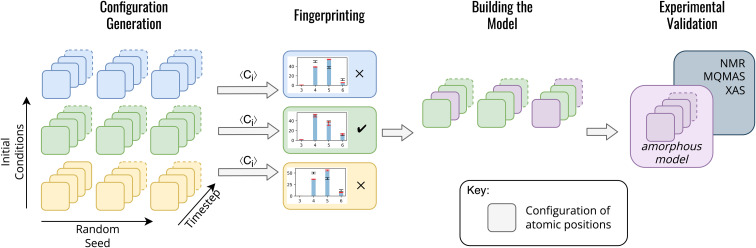
Method for generating an amorphous model from a series of static configurations from AIMD. Configuration generation: each colored box represents a single static configuration from an AIMD simulation, and their depth into the page represents the number of configurations over time (timestep arrow). These configurations are colored by their initial conditions (temperature, density, and rate of equilibration) such that blue, green, and yellow are three different sets of initial conditions. The three repeated sets of configurations represent repeated AIMD simulations with different randomly seeded starting points. In this schematic example there are 3 initial conditions and 3 randomly seeded AIMD simulations for each set of initial conditions. Fingerprinting: the results of simulations for each set of initial conditions are averaged across the final equilibrated 1000 AIMD steps of the simulation, and the ratios of the coordination environments are compared between the simulation and experiment. Averages over configurations which match experimental data are then used for building the model. The model consists of a total of 45 randomly selected configurations (15 from each individual AIMD simulation out of the 3 randomly seeded AIMD simulations). This model is collated and shown as the purple squares labeled ‘amorphous model’. These 45 configurations are then used in the experimental validation in which the total observable is calculated as an average over the static configurations in the amorphous model.

In contrast to previous methods for modelling am-Al_2_O_3,_ our method, outlined in Fig. 1, is both specific to a single experimental setup and combines configurations from across AIMD simulations. Both the DFT-calculated NMR and XAS spectra for our model match experimental results for ALD deposited alumina. Rather than choosing one configuration from AIMD, we select a range of static configurations which, when combined, contain local orderings that are representative of ALD deposited am-Al_2_O_3_.

To build this model, we first generate a large set of AIMD simulations using a melt-quench technique which span a range of initial conditions (densities, equilibration temperatures, and rate of equilibration) as outlined in Fig. 1 configuration generation. For each set of initial conditions, the AIMD simulations are repeated from different starting structures, thereby exploring additional local structural orderings. For the am-Al_2_O_3_ model, we generated 18 initial conditions, with 3 starting structures of 120 atoms each to generate a total of 54 000 static configurations. A 120-atom model was used for each AIMD simulation to allow for computational efficiency, given the computational intensity of both NMR and XAS in DFT. We simulated am-Al_2_O_3_ at densities of 3.18, 3.30, and 3.42 g cm^−3^, equilibration temperatures or 300, 600, and 900 K, and using two different equilibration rates (the combination of these parameters results in 18 distinct initial conditions).

Each set of initial conditions was chosen based on literature of am-Al_2_O_3_ to narrow our starting search space. The density range was chosen based on previous experimental literature on am-Al_2_O_3_ which suggests a range of possible densities between 3.05 to 3.40 g cm^−3^.^56,59^ Although models at lower densities, such as the Lizárraga *et al.* 2.9 g cm^−3^ model, exist, such low density models often contain AlO_3_ environments.^26,60^ The ^27^Al NMR and Al K-edge XAS on am-Al_2_O_3_ both in this manuscript and in the literature on ^27^Al NMR,^61,62^ shows no clear evidence for threefold coordinated Al environments, and therefore we chose to exclude any low density models which would promote AlO_3_ formation. The two equilibration rates, which we refer to as a ‘cooling’ and ‘quenching’, are in line with two distinct schemes of amorphous model generation found in the literature.^29,56^ The ‘cooling’ method is more commonly used,^26,56^ as slower cooling rates have been shown to give better results in line with experiment, however the ‘quenching’ method used on a slab model of am-Al_2_O_3_ and Al resulted in an am-Al_2_O_3_ model with similar RDFs to experiment,^29^ prompting us to also explore this scheme.

For each initial condition, 120 atoms with stoichiometry Al_2_O_3_ were packed into a cubic simulation cell, using Packmol.^63^ The cell was then melted at a temperature of 4000 K for 10 ps (5000 AIMD steps with a 2 fs time step). In the ‘cooling’ scheme, the cell was then cooled over 10 ps to the desired equilibration temperature using the Nose–Hoover thermostat, whereas when using the ‘quenching’ scheme, the cell was immediately equilibrated from the melt for a further 10 ps.

The radial distribution functions (RDF) from the final 1000 timesteps of each equilibration from AIMD was compared to the Lamparter *et al.* RDF^30^ in the ESI Fig. S3.† Interestingly, a majority of our models each with different densities, equilibration temperatures, and cooling schemes have comparable RDFs with experiment, demanding a further narrowing of this initial set of data to obtain a model which is specific to the experimental ALD deposited am-Al_2_O_3_. This striking similarity between RDFs for am-Al_2_O_3_ despite the range of initial conditions highlights a major advantage of our method over previous methods for modelling am-Al_2_O_3_ (and indeed many other amorphous structures). All previous am-Al_2_O_3_ models^26,28,55,56^ use RDF as the sole metric for verifying that the structure is comparable to experiment. However, the results highlighted in Fig. S3† indicate that the RDF alone is not a clear indicator of structural difference between models.

**Fig. 2:**
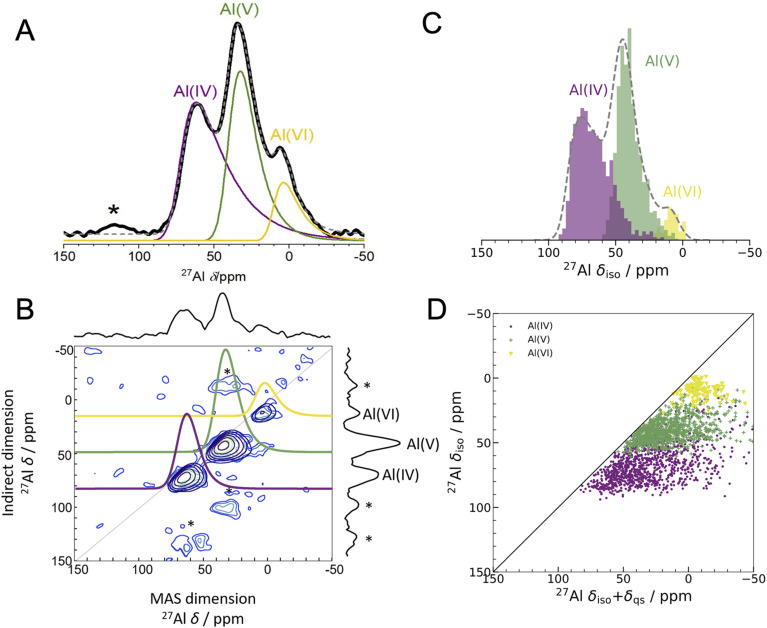
Experimental 1D NMR and MQMAS compared to DFT-calculated isotropic chemical shifts (A) 1D ^27^Al experimental NMR (black) at 16.44 T with three fits using the Czjzek model (solid lines; see Experimental methods and Table S3† for fitting parameters) a model commonly applied to spectra of quadrupolar nuclei such as ^27^Al when the materials are disordered.^41,42^ The fits are summed to produce overall 1D spectra (dashed grey) of ALD deposited am-Al_2_O_3_. Spinning sidebands are indicated with an asterisk. Each signal is colored according to the closest geometric environment, based on experimental shift values for Al(iv : v : vi). (B) MQMAS at 16.44 T of ALD deposited am-Al_2_O_3_ shows that Al(iv) and Al(v) both have large quadrupolar shifts in the MAS dimension. (C) GIPAW NMR DFT-calculated spectrum of am-Al_2_O_3_. The distributions of Al environments are shown as a histogram, with the total spectrum shown in grey and Gaussian broadened to guide the eye and allow comparison with panel (A). This is a histogram showing only the chemical shifts, *δ*_iso_, and neglecting the effects of the second-order quadrupolar interaction (shown in Fig. 4). (D) Comparison of *δ*_iso_ and *δ*_iso_ + *δ*_qs_ from DFT-calculated NMR where *δ*_qs_ is calculated using the method from Engelhardt.^41^ This method again shows distortions in the quadrupolar dimension for both Al(iv) and Al(v), which is consistent with the MQMAS in (B). The distribution of *δ*_iso_ + *δ*_qs_ indicates that quadrupolar effects play a large role in the experimental 1D lineshape.

**Fig. 3:**
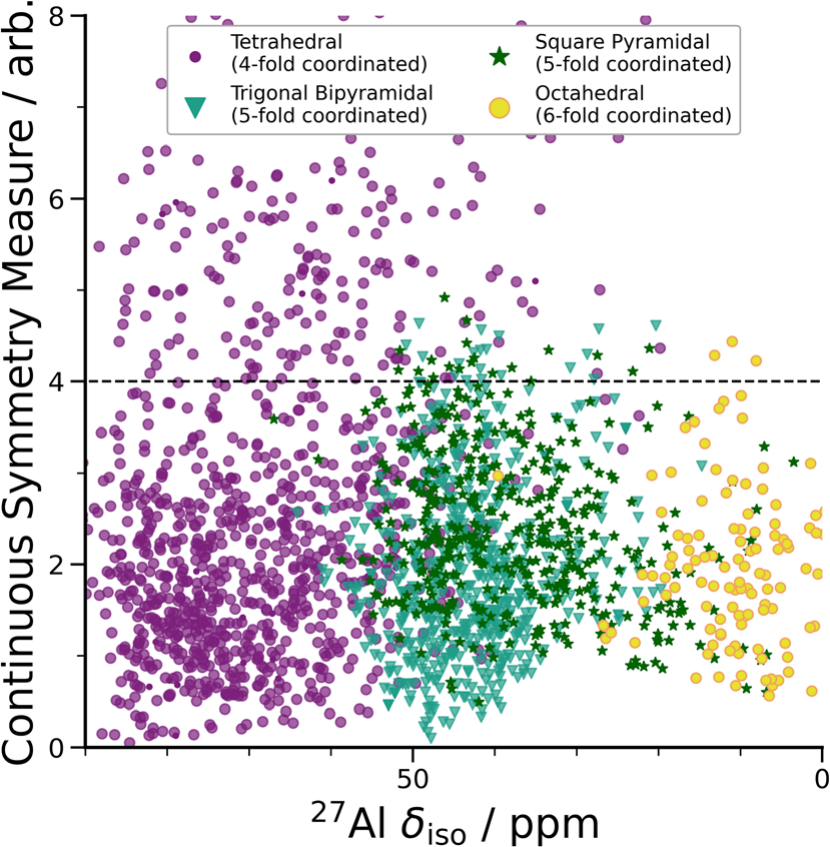
DFT-calculated isotropic chemical shifts compared to the distortion of its geometric environment. GIPAW NMR calculated *δ*_iso_*vs.* CSM as defined by ChemEnv.^70^ Each coordination environment is broken down into its closest geometric environment and coordination number: Al(iv) sites were classified as tetrahedral, Al(v) sites were subdivided into trigonal bipyramidal and square pyramidal and Al(vi) as octahedral. There are a large number of distorted (CSM > 4) tetrahedral environments, which have a range of *δ*_iso_ shifts.

**Fig. 4:**
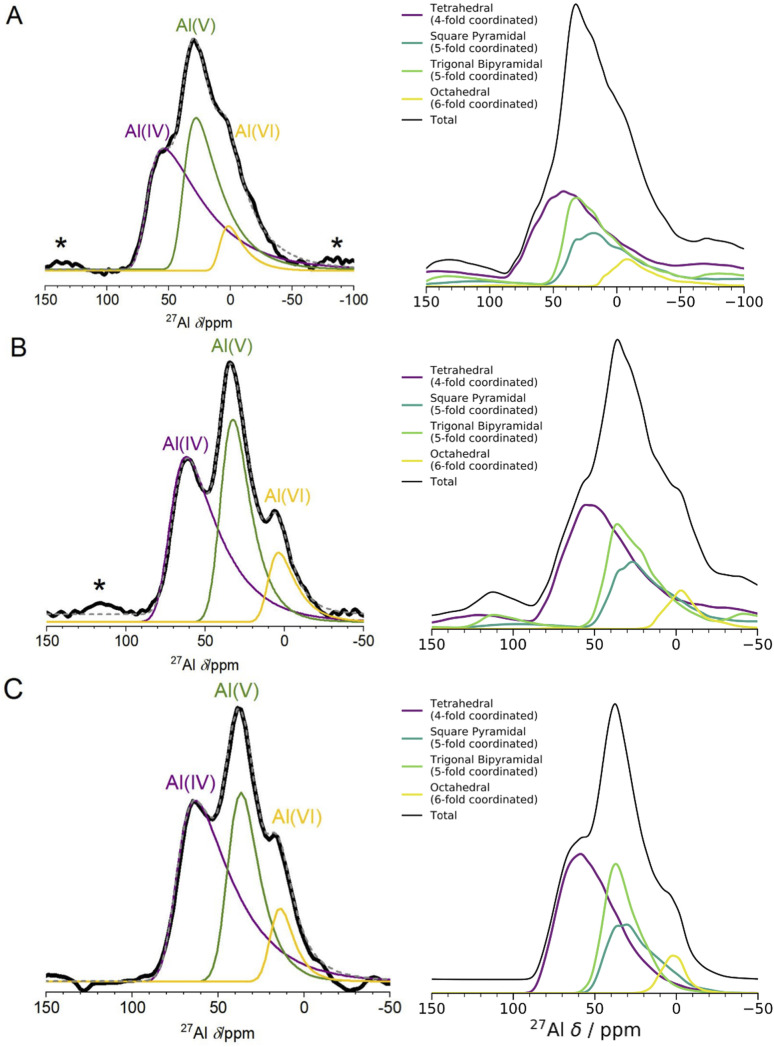
1D ^27^Al NMR experimental one pulse spectra at 3 fields and DFT computed spectra. The left panel shows experimental 1D NMR spectra measured at 3 different fields ((A) 11.75 T, (B) 16.44 T, (C) 23.49 T). The spectra in (A) and (B) were measured using a one pulse sequence, and the spectrum in (C) was measured using a Hahn-echo pulse sequence. Spinning sidebands are marked with an asterisk (*), and the fittings for each Al coordination environment were performed using the Czjzek model.^41,42^ The right panel shows the results of spin-simulations at the same 3 fields with quadrupolar lineshapes included using SIMPSON.^48^ DFT-calculated *δ*_iso_, *η*_Q_ and *C*_Q_ were used as input for the spin-simulations. DFT-calculated spectra show Al(v) lineshapes separated into square pyramidal and trigonal bipyramidal environments. Although the DFT/SIMPSON calculated lineshapes are broader than the experimental lineshapes, we can see a trend from low to high field that the individual environments have sharper peaks. This is expected, as higher field NMR is able to better resolve individual contributions from quadrupolar nuclei.

We introduce a Fingerprinting step to identify a model which is specific to ALD deposited am-Al_2_O_3_ (Fig. 1). Rather than compared to the experimental RDF, we use the ratio of four- five- and six-fold Al environments (Al(iv), Al(v) and Al(vi)), extracted from the experimental ^27^Al NMR spectrum for ALD deposited am-Al_2_O_3_. The ratio of Al(iv : v : vi) within the ALD deposited am-Al_2_O_3_ are 50%, 38%, and 12% ± 2%, respectively (Fig. 2, 3, and S1, S2†). The concentrations of these coordination environments are extracted by fitting the one-dimensional (1D), one pulse ^27^Al NMR spectrum obtained at 16.44 T using the Czjzek model as described in the Experimental methods section and discussed in more detail below. Intensity ratios for the Al(iv : v : vi) coordinated environments of 51 : 41 : 8 and 56 : 34 : 10 were obtained at lower and higher fields respectively (Fig. 4 and Table S3†) suggesting that experimental errors are slightly larger than ±2%. We have, however, chosen to take the values determined from the intermediate field spectrum because this has the highest signal to noise and, unlike the high field spectrum, was acquired with a one-pulse spectrum.

All AIMD simulations that do not have concentrations of Al(iv : v : vi) within the experimental tolerances (±2%) are then excluded. What remains are AIMD simulations comprising configurations which capture the specific local environments of am-Al_2_O_3_; we designed this approach as a way to incorporate more informative experimental data to narrow down the configuration space of simulations to those that are most likely to capture the properties of ALD deposited am-Al_2_O_3_.

Of the 18 initial conditions considered, just two AIMD simulations have ratios of Al(iv : v : vi) within ±2% error of the experimental 1D NMR data. These are the set of simulations run at 600 K and 300 K using the ‘cooling’ scheme at a density of 3.18 g cm^−3^. The results for the 300 K model are shown throughout the main text and the 600 K results are shown in the ESI† with comparable resulting DFT-calculated spectra. The other 16 models, while also within the initial range of possible experimental conditions, do not compare well to our specific ALD coating of 1000 layers of am-Al_2_O_3_, but could possibly be used in future work to model other phases of am-Al_2_O_3_ with different ratios of Al coordination.

We construct the amorphous model as a set of randomly selected static configurations compiled from the AIMD simulations which met the Fingerprinting criteria. A set of 15 configurations across the final 1000 timesteps were randomly chosen from each of the 3 AIMD simulations for the 300 K 3.18 g cm^−3^ ‘cooling’ scheme model. The final amorphous model is the set of the 45 randomly selected configurations. Calculations of material properties are then obtained as an unweighted average over these 45 configurations. Finally, a check was added to ensure that the 45 randomly selected configurations retained the same coordination environment ratios as the original Fingerprinting step.

### DFT-calculated spectroscopy on am-Al_2_O_3_

For some observable property, *X*, such as the NMR chemical shift or XAS absorption energies, we can calculate its average value across a set of static configurations (*N*_c_) as,
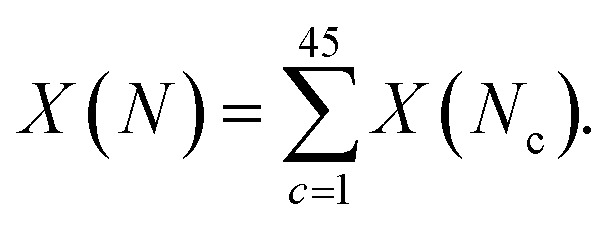


This is analogous to an ensemble average of the property in which the probability of each configuration is equally likely, as is the case for an amorphous solid with no symmetry equivalence. The ensemble average NMR and XAS spectra were calculated across the 45 configurations of am-Al_2_O_3_ in our model, and compared to experimental results from the 1000 layer sample of ALD deposited am-Al_2_O_3_. Reproducing these experimental signatures indicates that this model captures the local order, and electronic properties of the experimental amorphous phase.

The experimental ^27^Al 1D NMR spectrum shown in Fig. 2A has 3 distinct peaks corresponding to disordered Al(iv), Al(v) and Al(vi) Al coordination environments present in a ratio of approximately 50%, 38%, and 12% ± 2% respectively, as determined from the fits to the 1D NMR 16.44 T spectrum shown in Fig. 2A. The presence of the Al(v) signal at 41 ppm and the low intensity of the Al(vi) signal at 11 ppm are strong indicators of the amorphous nature,^64,65^ and the Al(iv) signal at 74 ppm is also characteristic of am-Al_2_O_3_. It is expected that a distribution of distorted Al(iv : v : vi) environments, combined with the quadrupolar nature of the ^27^Al nucleus, would lead to large quadrupolar coupling constants and a wide range of isotropic chemical shifts. This is confirmed by the broad asymmetric peaks shown in the 1D NMR spectrum in Fig. 2A and in all three spectra in Fig. 4. Fits to the 1D spectrum using a Czjzek model capture the distribution in *C*_Q_ by fitting the spectra with a Gaussian distribution in the values of the three orthogonal components that describe the electric field gradient tensor. The average magnitudes for *C*_Q_, 〈|*C*_Q_|〉, were extracted using the Czjzek model as 10.9, 7.8 and 7.9 MHz for the Al(iv), (v) and (vi)-coordinated sites, respectively, at a field of 16.4 T (Table S3†). Within the Czjzek model, the standard deviation, *σ*, for the distribution of values of *C*_Q_ is approximately 〈|*C*_Q_|〉/1.995. For the Al(iv) site, *σ* is 5.5 MHz and *C*_Q_ ranges from zero to above 20 MHz.^66^ The experimental 2D MQMAS (multi quantum MAS) NMR (Fig. 2B) contains three signals spreading along the isotropic diagonal line and horizontally along the MAS dimension, also highlighting the spread of quadrupolar coupling constants and a chemical shift distribution in the experimental am-Al_2_O_3_ sample.

The experimental quantification of ^27^Al NMR is known to be a challenging task due to the presence of large quadrupolar parameters and highly distorted Al sites which lead to broadened signals from NMR.^61,67^ Further details of the experimental fitting parameters from Fig. 2 and 4 can be found in the ESI section S2.† In addition, a five-component fit was constructed, to account for “NMR invisible” Al components in the 3QMAS experiment (compared to the 1D NMR) at 16.44 T and is shown in Fig. S6.†

The DFT-calculated NMR isotropic chemical shifts for each configuration in the amorphous model reproduce the total isotropic range and location of shifts in the experimental 1D spectrum (Fig. 2C). The 1D NMR has three main peaks, identified by the Al(iv : v : vi) environments, with *δ*_iso_ at 74, 45, and 10 ppm in DFT and 74, 41, and 11 ppm in the experiment shown in Fig. 2A. In addition, the calculated quadrupolar induced shifts shown in Fig. 2D have wide distributions especially in the Al(iv) and Al(v) sites. The calculated *δ*_qs_ shift is primarily influenced by the magnitude of *C*_Q_, indicating a large distribution of quadrupolar coupling constants. The DFT-calculated *C*_Q_ values for Al(iv) sites ranges from zero to 25 MHz, and for Al(v) sites is zero to 20 MHz (shown in Fig. S7†), while for Al(vi) sites the range is smaller from zero to 15 MHz. A similar distribution of high *C*_Q_ values for Al(iv) and Al(v) sites is found in both aluminosilicate glasses and sol–gel prepared MgO–Al_2_O_3_ and ZrO_2_–Al_2_O_3_.^68,69^

A wide range of *C*_Q_ values is an indicator of distorted Al environments within the sample. One advantage of calculating the NMR spectrum using DFT, is that the chemical shift tensor is calculated for each atom in the model. We can therefore construct spectra based on atom-specific coordination environments. Using a continuous-symmetry measure (CSM),^70^ a measure of the relative distortion of each Al environment from its closest geometric environment was extracted for each site in the model. A comparison of the CSM to isotropic chemical shift (Fig. 3), shows that most Al(iv) sites are distorted from a tetrahedral geometry (CSM > 4).^70^ Distorted sites give rise to larger *C*_Q_ values, and therefore this wide range of distorted Al(iv) sites is in agreement with the wide range (zero to 25 MHz) of the DFT-calculated *C*_Q_ values for Al(iv).

Separating the DFT-calculated 1D NMR spectrum into the closest geometric environment, as shown in Fig. 3, determines that two types of Al(v) environments, square pyramidal and trigonal bipyramidal, exist within the am-Al_2_O_3_ model. The signal from these two sites combine to form the Al(v) peak in the 1D NMR at 41 ppm, with a range of site-specific shifts from 20 to 60 ppm. Both these Al(v) sites are less distorted, on average, than the Al(iv) sites reflecting their slightly smaller distributions in *C*_Q_ values. There is limited experimental literature on the two geometric Al(v) environments, as their spectra are not easily deconvoluted from (experimental) 1D ^27^Al NMR.^62^ The model of am-Al_2_O_3_ from DFT enables the construction of these two sites' spectra, while their overlapping ^27^Al *δ*_iso_ values results in one single Al(v) assignment in the experimental 1D spectrum.

The spin-simulation tool SIMPSON^48^ was used to incorporate quadrupolar interactions into the DFT-calculated shifts at three fields (11.75 T, 16.44 T, 23.49 T) corresponding to the three fields at which the experimental NMR spectra (Fig. 4 left panel) were obtained. The resulting DFT-calculated spectra are shown in Fig. 4 (right panel) and the distribution of chemical shifts for the Al(iv) and Al(v) environments is well described. Summing together the individual spectra for each Al environment in the model results in environments with high *C*_Q_ values and low intensities leading to a long sloping tail in the resulting Al(iv) and Al(v) environments' total spectrum. This sloping tail is a feature explicitly incorporated into the Czjzek model which was used to carry out the experimental fittings for each environment. However, the DFT-calculated chemical shifts, asymmetry parameters, and quadrupolar coupling constants which are used to produce the spectra shown in Fig. 4 (right panel) also have this same shape, indicating that the Al environments in the model are similar to those expected for amorphous systems with quadrupolar nuclei. In addition, by incorporating the magnetic-field strength into the simulation, and comparing with the experimental results at three fields, we find that higher fields show sharper peaks of the Al(iv : v : vi) environments, and a narrower overall spectral width. This is more pronounced in the experimental results, which may be partly a result of the DFT-calculated *C*_Q_ values being overestimated, leading to a broadening in the NMR peaks. This overestimation is documented in the literature on DFT-calculated EFG tensors^61,71^

**Fig. 5:**
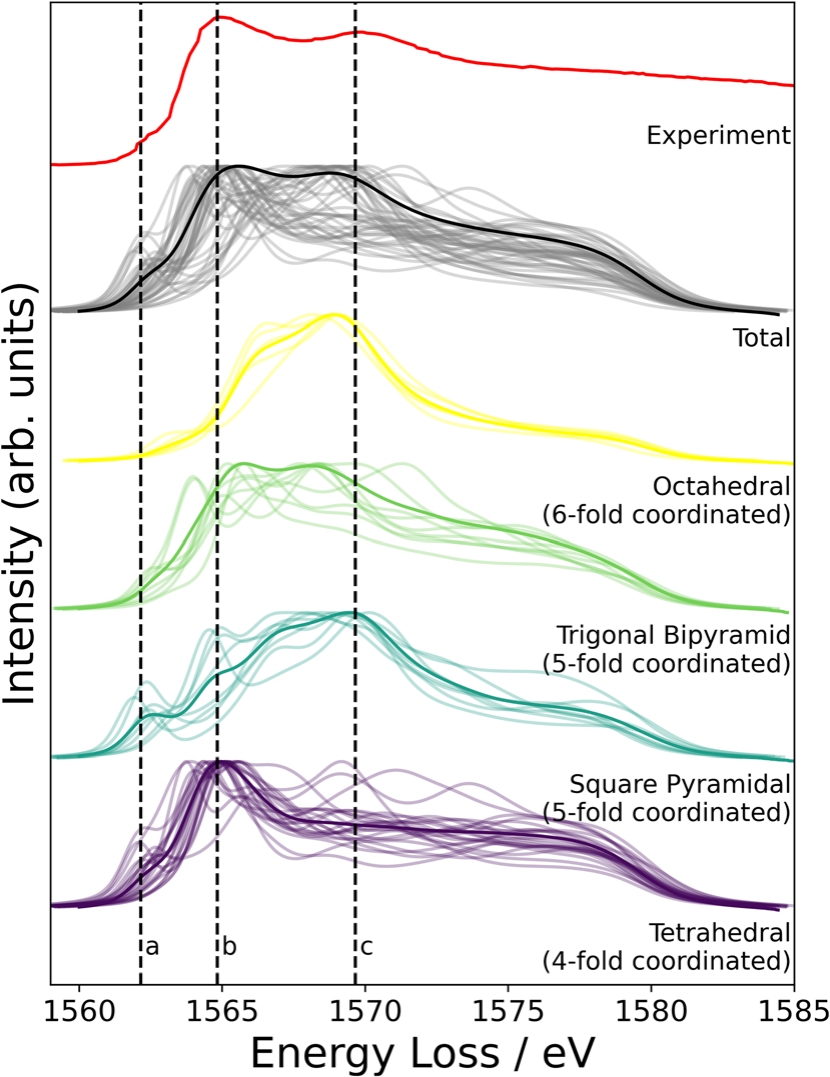
Experimental XAS spectrum obtained from the ALD deposited sample compared to the calculated core-hole XAS. The experimental spectrum is shown in red, and is obtained from the 1000 layer ALD deposited sample (details in Experimental methods section). Three distinct features at 1562, 1565, and 1570 eV are denoted by dashed lines a, b, and c. The grey lines in the total spectrum from DFT show each individual spectra calculated at a single Al site from the am-Al_2_O_3_ model, and the solid black line is the sum of those spectra. DFT-calculated spectra separated by coordination environment are shown, colored by coordination environment; thin lines are individual spectra, thick lines are the sum for each environment. All Al(iv) sites were classified as tetrahedral, Al(v) sites were subdivided into trigonal bipyramidal and square pyramidal, and Al(vi) as octahedral.

NMR probes local atomic structure, while XAS probes the local electronic structure, of am-Al_2_O_3_. The experimental Al K-edge XAS spectrum shown in Fig. 5, exhibits three main features; a pre-edge feature (a) and two dominant broad peaks at 1565 eV (b) and 1570 eV (c) which are similar to those in Al-rich glasses^72^ and attributed to transitions in Al(iv) and Al(vi) respectively. The absorption edge for Al(v) lies between Al(iv) and Al(vi), and has no experimental reference. Calculating core-hole spectra for all Al sites in the amorphous model, determines the location of this Al(v) absorption edge between 1565 and 1570 eV (Fig. 5), and confirms the absorption energy of the Al(iv) and Al(vi) peaks, indicating that the model's electronic structure is consistent with that of experimental am-Al_2_O_3_.

In addition to identifying the two main XAS peaks, the pre-peak at 1562.5 eV was also reproduced using DFT-calculated XAS. Previous experimental work on Al K-edge spectroscopy in zeolites also identifies a pre-edge at 1563 eV,^73^ which they attribute to transitions in Al(iii) sites. A pre-edge at 1565 eV is also documented for the crystalline α-Al_2_O_3_ phase, and assigned to 1s to 3s transitions in Al(vi) sites.^74,75^ The pre-edge in am-Al_2_O_3_ is at 1562.5 eV, both in experiment and in the DFT-calculated spectrum, as shown in Fig. 5, and occurs in either tetrahedral Al(iv) sites or square pyramidal Al(v) sites. DFT-calculated spectra for individual Al(iv) sites in our am-Al_2_O_3_ model show that the pre-edge at 1562.5 eV is a result of transitions from Al 1s to 3s states, within distorted Al environments. This transition is expected in distorted Al(iv) or Al(v) sites where the local centrosymmetry is broken, and transitions below the main absorption energy may occur. Because the configurations generated in the model of am-Al_2_O_3_ presented in this work were obtained from AIMD at 300 K, atomic vibrations at that temperature are naturally included in the model without the need to calculate additional dynamical effects.^74^ As the Al(v) main peak at b (1565 eV in Fig. 5) is at a similar absorption energy as the Al(iv) peak, the Al(v) contributions are not separated in experiment, but are easily distinguished using XAS calculated from DFT. While such deconvolution is routinely applied in crystalline systems,^76–79^ this result demonstrates the capability of performing a similar analysis on an amorphous material.

**Fig. 6:**
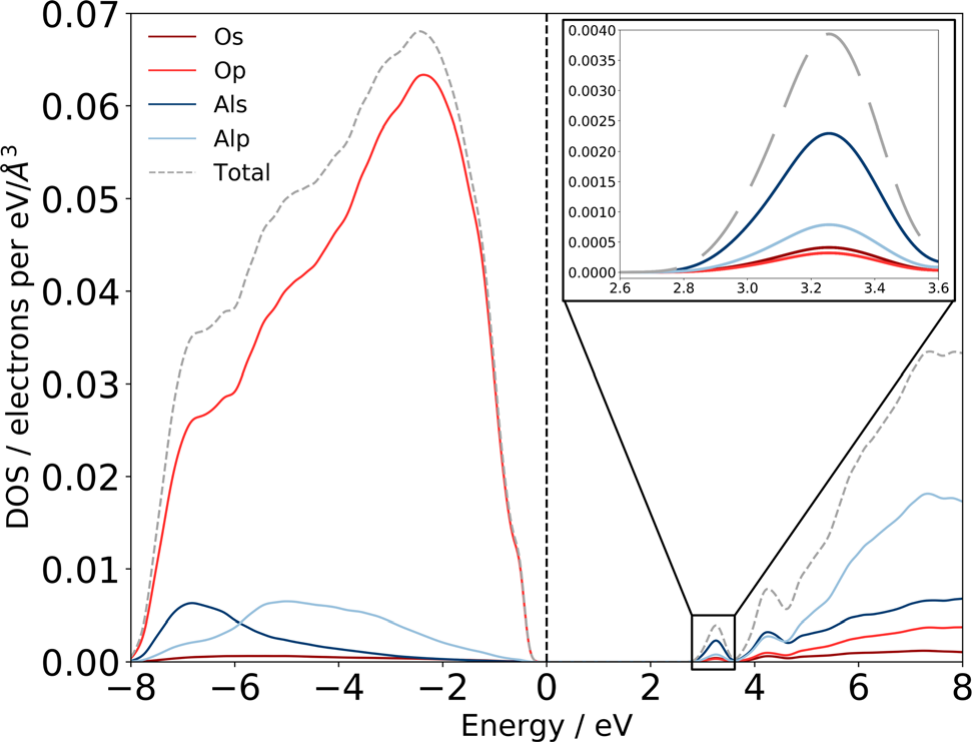
Computed eDOS for amorphous alumina. Total eDOS separated by atom and orbital contribution shows that am-Al_2_O_3_ is a wide bandgap insulator, within PBE with a gap of 2.6 eV. States at the top of the valence band, near the Fermi level are primarily O p states, with states in the conduction-band minimum being primarily Al s (detail shown inset top right). The dashed grey line is the sum of these states, and the total eDOS was broadened using a Gaussian function of width 0.1 eV, as implemented in OptaDOS.^51,52^ The Fermi level is set to 0 eV for all configurations. Two localized states at 3.2 and 4.2 eV above the Fermi level have Al s character and mixed Al s, p character, respectively.

Previous experimental XAS on the Al L_2,3_-edge^80^ proposes that the location of the conduction band minimum (CBM) is governed by the charge transfer from Al to O atoms, specifically between the O 2p states at the valence band maximum (VBM) and Al 3s states at the CBM. We can confirm the experimental assignments of the orbital character with the electronic structure of our am-Al_2_O_3_ model by calculating the eDOS as an unweighted average across the 45 configurations, as shown in Fig. 6. States at the VBM are O p type character, and states at the CBM are Al s character. Interestingly, we identify two small peaks at the bottom of the conduction band at 3.2 and 4.2 eV which are low density states in this material. The computed eDOS also confirms the experimentally predicted wide band-gap insulating nature of am-Al_2_O_3_.^76–79^

## Conclusions

Previous work^3,81^ implies that amorphous models require thousands of atoms in the unit cell or semi-infinite simulation times to fully capture local properties. We show that by averaging across AIMD simulations and incorporating experimental insight into the sampling approach, we produce a model of am-Al_2_O_3_ which exhibits the same local structural properties as captured by experimental NMR and the same electronic properties as captured by XAS.

Identifying a subset of configurations from AIMD to generate the model of am-Al_2_O_3_, enables the calculation of electronic properties, such as the eDOS, NMR and XAS, which is not possible using classical simulation methods. This is especially important for functional materials such as alumina, which are routinely used in electronic devices. We further demonstrate the importance of incorporating experimental insight at the model configuration sampling stage, as we describe a set of 54 different AIMD simulations, all with sensible initial conditions and RDFs, but only two of which with coordination environments within the experimental margin of error. This additionally underscores the need for having a specific experimental comparison metric when generating amorphous models; the results presented in this work are all with reference to one ALD deposited sample of alumina using the most relevant possible spectroscopy methods for characterization.

This article illustrates the importance of the connection between choosing the relevant metrics (here coordination environment) for developing a model and calculating experimental observables. The method of averaging across static configurations from experimentally informed sampling of configurations from AIMD simulations can be applied to other amorphous systems, where local structure–property relationships are critical to the accurate modelling of the system. For example, in amorphous VS_4_, experimental ^51^V NMR and S K-edge XANES show structural transformations during Li-ion battery cycling,^82^ which could be modeled using the presented method. More broadly, the class of amorphous metal oxides (among them MnO_2_, Fe_2_O_3_, TiO_2_, and a host of others) are used in energy storage applications, and their local structure and ordering of the metal oxide coordination environments plays a large role in the function and optimization of energy storage devices.^83,84^ We hope that this work has shed light on the importance of choosing the appropriate parameters for modelling amorphous materials, and offered a new perspective on utilizing experimental spectroscopy in the modelling of amorphous phases from first principles.

## Data availability

The computational data including structures, NMR, and XAS spectra, as well as the electronic DOS found in this article are published online in the Cambridge Data Repository at https://doi.org/10.17863/CAM.92274.

## Author contributions

AF Harper: formal analysis, writing – original draft preparation, data curation, conceptualization. SE Emge: investigation, writing – review and editing. PCMM Magusin: support with MQMAS setup and analysis, organization of 1 GHz measurements. CP Grey: supervision, resources, writing – review and editing. AJ Morris: supervision, resources, writing – review and editing.

## Conflicts of interest

There are no conflicts to declare.

## Supplementary Material

SC-014-D2SC04035B-s001

## References

[cit1] Hoja J., Ko H.-Y., Neumann M. A., Car R., DiStasio Jr R. A., Tkatchenko A. (2019). Reliable and Practical Computational Description of Molecular Crystal Polymorphs. Sci. Adv..

[cit2] Woodley S., Catlow R. (2008). Crystal Structure Prediction from First Principles. Nat. Mater..

[cit3] Sivaraman G., Krishnamoorthy A. N., Baur M., Holm C., Stan M., Csányi G., Benmore C., Vázquez-Mayagoitia Á. (2020). Machine-Learned Interatomic Potentials by Active Learning: Amorphous and Liquid Hafnium Dioxide. npj Comput. Mater..

[cit4] Deringer V. L., Bernstein N., Csányi G., ben Mahmoud C., Ceriotti M., Wilson M., Drabold D. A., Elliott S. R. (2021). Origins of Structural and Electronic Transitions in Disordered Silicon. Nature.

[cit5] Aykol M., Dwaraknath S. S., Sun W., Persson K. A. (2018). Thermodynamic Limit for Synthesis of Metastable Inorganic Materials. Sci. Adv..

[cit6] Das C., Kot M., Hellmann T., Wittich C., Mankel E., Zimmermann I., Schmeisser D., Khaja Nazeeruddin M., Jaegermann W. (2020). Atomic Layer-Deposited Aluminum Oxide Hinders Iodide Migration and Stabilizes Perovskite Solar Cells. Cell Rep. Phys. Sci..

[cit7] Yang Z., Albrow-Owen T., Cui H., Alexander-Webber J., Gu F., Wang X., Wu T. C., Zhuge M., Williams C., Wang P., Zayats A. v., Cai W., Dai L., Hofmann S., Overend M., Tong L., Yang Q., Sun Z., Hasan T. (2019). Single-Nanowire Spectrometers. Science.

[cit8] Lu J., Fu B., Kung M., Xiao G., Elam J., Kung H., Stair P. (2012). Coking- and Sintering-Resistant Palladium Catalysts Achieved through Atomic Layer Deposition. Science.

[cit9] Fukuhara M., Kuroda T., Hasegawa F., Hashida T., Takeda M., Konno K., Fujima N. (2021). AlO_6_ Clusters' Electric Storage Effect in Amorphous Alumina Supercapacitors. Sci. Rep..

[cit10] Kim M. G., Kanatzidis M. G., Facchetti A., Marks T. J. (2011). Low-Temperature Fabrication of High-Performance Metal Oxide Thin-Film Electronics via Combustion Processing. Nat. Mater..

[cit11] Yang Y., Zhou J., Zhu F., Yuan Y., Chang D. J., Kim D. S., Pham M., Rana A., Tian X., Yao Y., Osher S. J., Schmid A. K., Hu L., Ercius P., Miao J., Yuan Y. (2021). Determining the Three-Dimensional Atomic Structure of an Amorphous Solid. Nature.

[cit12] Bhattarai B., Pandey A., Drabold D. A. (2018). Evolution of Amorphous Carbon across Densities: An Inferential Study. Carbon.

[cit13] Cernuto G., Galli S., Trudu F., Colonna G. M., Masciocchi N., Cervellino A., Guagliardi A. (2011). Investigating the Amorphous–Crystalline Interplay in SiO_2_/TiO_2_ Nanocomposites by Total Scattering Methods. Angew. Chem., Int. Ed..

[cit14] Pedersen A., Pizzagalli L., Jónsson H. (2017). Optimal Atomic Structure of Amorphous Silicon Obtained from Density Functional Theory Calculations. New J. Phys..

[cit15] Caro M. A., Aarva A., Deringer V. L., Csányi G., Laurila T. (2018). Reactivity of Amorphous Carbon Surfaces: Rationalizing the Role of Structural Motifs in Functionalization Using Machine Learning. Chem. Mater..

[cit16] Deringer V. L., Csányi G. (2017). Machine Learning Based Interatomic Potential for Amorphous Carbon. Phys. Rev. B.

[cit17] Zhou Y., Kirkpatrick W., Deringer V. L., Zhou Y., Kirkpatrick W., Deringer V. L. (2021). Cluster Fragments in Amorphous Phosphorus and Their Evolution under Pressure. Adv. Mater..

[cit18] Artrith N., Urban A., Ceder G. (2018). Constructing First-Principles Phase Diagrams of Amorphous LixSi Using ML Assisted Sampling. J. Chem. Phys..

[cit19] Paruzzo F. M., Hofstetter A., Musil F., De S., Ceriotti M., Emsley L. (2018). Chemical Shifts in Molecular Solids by Machine Learning. Nat. Commun..

[cit20] Torrisi S. B., Carbone M. R., Rohr B. A., Montoya J. H., Ha Y., Yano J., Suram S. K., Hung L. (2020). Random Forest Machine Learning Models for Interpretable X-Ray Absorption near-Edge Structure Spectrum-Property Relationships. npj Comput. Mater..

[cit21] Levin I., Brandon D. (2005). Metastable Alumina Polymorphs: Crystal Structures and Transition Sequences. J. Am. Ceram. Soc..

[cit22] Vinod Chandran C., Kirschhock C. E. A., Radhakrishnan S., Taulelle F., Martens J. A., Breynaert E. (2019). Alumina: Discriminative Analysis Using 3D Correlation of Solid-State NMR Parameters. Chem. Soc. Rev..

[cit23] Peintinger M. F., Kratz M. J., Bredow T. (2014). Quantum-Chemical Study of Stable, Meta-Stable and High-Pressure Alumina Polymorphs and Aluminum Hydroxides. J. Mater. Chem. A.

[cit24] Sarou-Kanian V., Gleizes A. N., Florian P., Samélor D., Massiot D., Vahlas C. (2013). Temperature-Dependent 4-, 5- and 6-Fold Coordination of Aluminum in MOCVD-Grown Amorphous Alumina Films: A Very High Field ^27^Al-NMR Study. J. Phys. Chem. C.

[cit25] Lee S. K., Ahn C. W. (2014). Probing of 2 Dimensional Confinement-Induced Structural Transitions in Amorphous Oxide Thin Film. Sci. Rep..

[cit26] Lizárraga R., Holmström E., Parker S. C., Arrouvel C. (2011). Structural Characterization of Amorphous Alumina and Its Polymorphs from First-Principles XPS and NMR Calculations. Phys. Rev. B: Condens. Matter Mater. Phys..

[cit27] van Hoang V., Kun Oh S. (2004). Simulation of Structural Properties and Structural Transformation of Amorphous Al_2_O_3_. Phys. B.

[cit28] Gutiérrez G., Johansson B. (2002). Molecular Dynamics Study of Structural Properties of Amorphous Al_2_O_3_. Phys. Rev. B: Condens. Matter Mater. Phys..

[cit29] Aykol M. and Persson K. A., Oxidation Protection with Amorphous Surface Oxides: Thermodynamic Insights from Ab Initio Simulations on Aluminum, 2018, 10.1021/acsami.7b14868 29297220

[cit30] Lamparter P., Kniep R. (1997). Structure of Amorphous Al_2_O_3_. Phys. B.

[cit31] Puurunen R. L. (2005). Surface Chemistry of Atomic Layer Deposition: A Case Study for the Trimethylaluminum/Water Process. J. Appl. Phys..

[cit32] George S. M. (2009). Atomic Layer Deposition: An Overview. Chem. Rev..

[cit33] Zhao Y., Amirmaleki M., Sun Q., Zhao C., Codirenzi A., Goncharova L. v., Wang C., Adair K., Li X., Yang X., Zhao F., Li R., Filleter T., Cai M., Sun X. (2019). Natural SEI-Inspired Dual-Protective Layers via Atomic/Molecular Layer Deposition for Long-Life Metallic Lithium Anode. Matter.

[cit34] Lotfabad E. M., Kalisvaart P., Cui K., Kohandehghan A., Kupsta M., Olsen B., Mitlin D. (2013). ALD TiO_2_ Coated Silicon Nanowires for Lithium Ion Battery Anodes with Enhanced Cycling Stability and Coulombic Efficiency. Phys. Chem. Chem. Phys..

[cit35] Zhao Y., Goncharova L. V., Lushington A., Sun Q., Yadegari H., Wang B., Xiao W., Li R., Sun X. (2017). Superior Stable and Long Life Sodium Metal Anodes Achieved by Atomic Layer Deposition. Adv. Mater..

[cit36] Chang C.-Y., Lee K.-T., Huang W.-K., Siao H.-Y., Chang Y.-C. (2015). High-Performance, Air-Stable, Low-Temperature Processed Semitransparent Perovskite Solar Cells Enabled by Atomic Layer Deposition. Chem. Mater..

[cit37] Gao Z., Qin Y. (2017). Design and Properties of Confined Nanocatalysts by Atomic Layer Deposition. Acc. Chem. Res..

[cit38] O'Neill B. J., Jackson D. H. K., Lee J., Canlas C., Stair P. C., Marshall C. L., Elam J. W., Kuech T. F., Dumesic J. A., Huber G. W. (2015). Catalyst Design with Atomic Layer Deposition. ACS Catal..

[cit39] Chan J. C. C., Eckert H. (2001). High-Resolution ^27^Al-^19^F Solid-State Double Resonance NMR Studies of AlF_3_-BaF_2_-CaF_2_ Glasses. J. Non-Cryst. Solids.

[cit40] Massiot D., Fayon F., Capron M., King I., St' S., le Calvé S., Calvé C., Alonso B., Durand J.-O., Bujoli B., Gan Z., Hoatson G. (2002). Modelling One-and Two-Dimensional Solid-State NMR Spectra. Magn. Reson. Chem..

[cit41] Engelhardt G., Koller H. (1991). A Simple Procedure for the Determination of the Quadrupole Interaction Parameters and Isotropic Chemical Shifts from Magic Angle Spinning NMR Spectra of Half-Integer Spin Nuclei in Solids. Magn. Reson. Chem..

[cit42] Czjzek G., Fink J., Gotz F., Schmidt H., Coey J. M. D., Rebouillat J. P., Lienard A. (1981). Atomic Coordination and the Distribution of Electric Field Gradients in Amorphous Solids. Phys. Rev. B: Condens. Matter Mater. Phys..

[cit43] Kresse G., Furthmüller J. (1996). Efficient Iterative Schemes for *Ab Initio* Total-Energy Calculations Using a Plane-Wave Basis Set. Phys. Rev. B: Condens. Matter Mater. Phys..

[cit44] Evans D. J., Holian B. L. (1998). The Nose–Hoover Thermostat. J. Chem. Phys..

[cit45] Blöchl P. E. (1994). Projector Augmented-Wave Method. Phys. Rev. B: Condens. Matter Mater. Phys..

[cit46] Clark S. J., Segall M. D., Pickard C. J., Hasnip P. J., Probert M. I. J., Refson K., Payne M. C. (2005). First Principles Methods Using CASTEP. Z. Kristallogr. - Cryst. Mater..

[cit47] Pickard C. J., Mauri F. (2001). All-Electron Magnetic Response with Pseudopotentials: NMR Chemical Shifts. Phys. Rev. B: Condens. Matter Mater. Phys..

[cit48] Bak M., Rasmussen J. T., Nielsen N. S. I. M. P. S. O. N. (2000). A General Simulation Program for Solid-State NMR Spectroscopy. J. Magn. Reson..

[cit49] Gao S.-P., Pickard C. J., Perlov A., Milman V. (2009). Core-Level Spectroscopy Calculation and the Plane Wave Pseudopotential Method. J. Phys.: Condens. Matter.

[cit50] Clark S. J., Segall M. D., Pickard C. J., Hasnip P. J., Probert M. I. J., Refson K., Payne M. C. (2005). First Principles Methods Using CASTEP. Z. fur Krist. - Cryst..

[cit51] Nicholls R. J., Morris A. J., Pickard C. J., Yates J. R. (2012). OptaDOS - a New Tool for EELS Calculations. J. Phys.: Conf. Ser..

[cit52] Morris A. J., Nicholls R. J., Pickard C. J., Yates J. R. (2014). OptaDOS: A Tool for Obtaining Density of States, Core-Level and Optical Spectra from Electronic Structure Codes. Comput. Phys. Commun..

[cit53] Yates J. R., Wang X., Vanderbilt D., Souza I. (2007). Spectral and Fermi Surface Properties from Wannier Interpolation. Phys. Rev. B: Condens. Matter Mater. Phys..

[cit54] Mizoguchi T., Tanaka I., Gao S.-P., Pickard C. J. (2009). First-Principles Calculation of Spectral Features, Chemical Shift and Absolute Threshold of ELNES and XANES Using a Plane Wave Pseudopotential Method. J. Phys.: Condens. Matter.

[cit55] Liu D., Guo Y., Lin L., Robertson J. (2013). First-Principles Calculations of the Electronic Structure and Defects of Al_2_O_3_. J. Appl. Phys..

[cit56] Momida H., Hamada T., Takagi Y., Yamamoto T., Uda T., Ohno T. (2006). Theoretical Study on Dielectric Response of Amorphous Alumina. Phys. Rev. B.

[cit57] Lizárraga R., Holmström E., Parker S. C., Arrouvel C. (2011). Structural Characterization of Amorphous Alumina and Its Polymorphs from First-Principles XPS and NMR Calculations. Phys. Rev. B: Condens. Matter Mater. Phys..

[cit58] Sarou-Kanian V., Gleizes A. N., Florian P., Samélor D., Massiot D., Vahlas C. (2013). Temperature-Dependent 4-, 5- and 6-Fold Coordination of Aluminum in MOCVD-Grown Amorphous Alumina Films: A Very High Field ^27^Al-NMR Study. J. Phys. Chem. C.

[cit59] Lee S. M., Cahill D. G., Allen T. H. (1995). Thermal Conductivity of Sputtered Oxide Films. Phys. Rev. B: Condens. Matter Mater. Phys..

[cit60] Mavrič A., Valant M., Cui C., Wang Z. M. (2019). Advanced Applications of Amorphous Alumina: From Nano to Bulk. J. Non-Cryst. Solids.

[cit61] Haouas M., Taulelle F., Martineau C. (2016). Recent Advances in Application of ^27^Al NMR Spectroscopy to Materials Science. Prog. Nucl. Magn. Reson. Spectrosc..

[cit62] Kenneth J. D., MacKenzie M., Smith E. (2002). 27Al NMR. Pergamon Mater. Ser..

[cit63] Martínez L., Andrade R., Birgin E. G., Martínez J. M. (2009). PACKMOL: A Package for Building Initial Configurations for Molecular Dynamics Simulations. J. Comput. Chem..

[cit64] Lee S. K., Park S. Y., Yi Y. S., Moon J. (2010). Structure and Disorder in Amorphous Alumina Thin Films: Insights from High-Resolution Solid-State NMR. J. Phys. Chem. C.

[cit65] Kaushik M., Leroy C., Chen Z., Gajan D., Willinger E., Mü Ller C. R., Fayon F., Massiot D., Fedorov A., Copéret C., Lesage A., Florian P. (2021). Atomic-Scale Structure and Its Impact on Chemical Properties of Aluminum Oxide Layers Prepared by Atomic Layer Deposition on Silica. Chem. Mater..

[cit66] Werner-Zwanziger U., Paterson A. L., Zwanziger J. W. (2020). The Czjzek Distribution in Solid-State NMR: Scaling Properties of Central and Satellite Transitions. J. Non-Cryst. Solids.

[cit67] Fenzke D., Freude D., Fröhlich T., Haase J. (1984). NMR Intensity Measurements of Half-Integer Quadrupole Nuclei. Chem. Phys. Lett..

[cit68] O'Dell L. A., Savin S. L. P., Chadwick A. v., Smith M. E. (2006). A ^27^Al, ^29^Si, 25 Mg and ^17^O NMR Investigation of Alumina and Silica Zener Pinned, Sol-Gel Prepared Nanocrystalline ZrO_2_ and MgO. Faraday Discuss..

[cit69] Ren J., Zhang L., Eckert H. (2014). Medium-Range Order in Sol–Gel Prepared Al_2_O_3_–SiO_2_ Glasses: New Results from Solid-State NMR. J. Phys. Chem. C.

[cit70] Waroquiers D., Gonze X., Rignanese G.-M., Welker-Nieuwoudt C., Rosowski F., Göbel M., Schenk S., Degelmann P., André R., Glaum R., Hautier G. (2017). Statistical Analysis of Coordination Environments in Oxides. Chem. Mater..

[cit71] Bonhomme C., Gervais C., Babonneau F., Coelho C., Pourpoint F., Azaïs T., Ashbrook S. E., Griffin J. M., Yates J. R., Mauri F., Pickard C. J. (2012). First-Principles Calculation of NMR Parameters Using the Gauge Including Projector Augmented Wave Method: A Chemists Point of View. Chem. Rev..

[cit72] Neuville D. R., Cormier L., Massiot D. (2004). Al Environment in Tectosilicate and Peraluminous Glasses: A ^27^Al MQ-MAS NMR, Raman, and XANES Investigation. Geochim. Cosmochim. Acta.

[cit73] van Bokhoven J. A., van der Eerden A. M. J., Koningsberger D. C. (2003). Three-Coordinate Aluminum in Zeolites Observed with In Situ X-Ray Absorption Near-Edge Spectroscopy at the Al K-Edge: Flexibility of Aluminum Coordinations in Zeolites. J. Am. Chem. Soc..

[cit74] Cabaret D., Brouder C. (2009). Origin of the Pre-Edge Structure at the Al K-Edge: The Role of Atomic Vibrations. J. Phys.: Conf. Ser..

[cit75] Altman A. B., Pemmaraju C. D., Alayoglu S., Arnold J., Booth C. H., Braun A., Bunker C. E., Herve A., Minasian S. G., Prendergast D., Shuh D. K., Tyliszczak T. (2017). Chemical and Morphological Inhomogeneity of Aluminum Metal and Oxides from Soft X-Ray Spectromicroscopy. Inorg. Chem..

[cit76] Podder J., Lin J., Sun W., Botis S. M., Tse J., Chen N., Hu Y., Li D., Seaman J., Pan Y. (2017). Iodate in Calcite and Vaterite: Insights from Synchrotron X-Ray Absorption Spectroscopy and First-Principles Calculations. Geochim. Cosmochim. Acta.

[cit77] Mao Y., Liang X. X., Zhao G. J., Song T. L. (2019). The Structural and Optical Properties of Ternary Mixed Crystals In_*x*_Ga_1−*x*_As with Zinc-Blende Structure by First-Principle Calculations. Phys. B.

[cit78] Bugnet M., Jaouen M., Mauchamp V., Cabioc’H T., Hug G. (2014). Experimental and First-Principles Investigation of the Electronic Structure Anisotropy of Cr_2_AlC. Phys. Rev. B: Condens. Matter Mater. Phys..

[cit79] Shen Y., Yang X., Bian Y., Liu S., Tang K., Zhang R., Zheng Y., Gu S. (2018). First Principles Study on the Structural Stability and Optoelectronic Properties of In_*x*_Ga_1−*x*_As Materials with Different Indium Component. Mater. Res. Express.

[cit80] Filatova E. O., Konashuk A. S. (2015). Interpretation of the Changing the Band Gap of Al_2_O_3_ Depending on Its Crystalline Form: Connection with Different Local Symmetries. J. Phys. Chem. C.

[cit81] Deringer V., Bernstein N., Bartók A., Cliffe M., Kerber R., Marbella L., Grey C., Elliott S., Csányi G. (2018). Realistic Atomistic Structure of Amorphous Silicon from Machine-Learning-Driven Molecular Dynamics. J. Phys. Chem. Lett..

[cit82] Shimoda K., Koganei K., Takeuchi T., Matsunaga T., Murakami M., Sakaebe H., Kobayashi H., Matsubara E. (2019). Structural Characterization of an Amorphous VS_4_ and Its Lithiation/Delithiation Behavior Studied by Solid-State NMR Spectroscopy. RSC Adv..

[cit83] Yan S., Abhilash K. P., Tang L., Yang M., Ma Y., Xia Q., Guo Q., Xia H., Yan S. H., Tang L. Y., Yang M., Ma Y. F., Xia Q. Y., Guo Q. B., Xia H. (2019). Research Advances of Amorphous Metal Oxides in Electrochemical Energy Storage and Conversion. Small.

[cit84] Han X., Wu G., Du J., Pi J., Yan M., Hong X. (2021). Metal and Metal Oxide Amorphous Nanomaterials towards Electrochemical Applications. Chem. Commun..

