# Supplementary material for: Modelling amorphous materials via a joint solid-state NMR and X-ray absorption spectroscopy and DFT approach: application to alumina
Source: Chem Sci. 2022 Dec 21;14(5):1155–67. doi: 10.1039/d2sc04035b (PMC9891381; doi:10.1039/d2sc04035b)
Supplement: SC-014-D2SC04035B-s001 [file SC-014-D2SC04035B-s001.pdf]

Electronic Supplementary Material (ESI) for Chemical Science.
This journal is © The Royal Society of Chemistry 2022

Supplementary Materials for

Modelling amorphous materials via a joint solid-state NMR and X-Ray absorption spectroscopy and DFT approach: application to alumina

A. F. Harper[1,*], S. P. Emge[2], P. C. M. M. Magusin[2,3], C. P. Grey[2], A. J. Morris[4,*]

[1]Theory of Condensed Matter, Cavendish Laboratory, University of Cambridge, J. J. Thomson Avenue, Cambridge CB3 0HE, U.K.

[2]Yusuf Hamied Department of Chemistry, University of Cambridge, Lensfield Road, Cambridge CB2 1EW, UK

[3]Institute for Life Sciences & Chemistry, Hogeschool Utrecht, Heidelberglaan 7, 3584 CS Utrecht, Netherlands

[4]School of Metallurgy and Materials, University of Birmingham, Edgbaston, Birmingham B15 2TT, U.K

*afh41@cam.ac.uk, a.j.morris.1@bham.ac.uk

Table of Contents

1. Results of *Fingerprinting* step in amorphous model generation
2. NMR Data (Experimental and Computational)
    a. Computation of NMR spectra
    b. Experimental NMR spectra and comparison with DFT results
    c. Choosing an NMR spectrum to compare to DFT results using MQMAS
    d. Experimental NMR Referencing and Background
3. XAS spectra and electronic states
4. Effect of distortions on the electronic states of alumina
5. Additional spectra for amorphous alumina model at 600K 3.18 g/cm^3
6. Assessing stability of the method
7. Supplementary References

1. Results of *Fingerprinting* step in amorphous model generation

Following the structure generation on our model generation sampling approach (see *Results: Amorphous model generation)* each set of simulations with the same initial conditions were assigned *fingerprints* according to their Al-coordination environments ranging from 3-6 coordinated (Al(III) to Al(VI). Fig. S1 and Fig. S2 show the fingerprints for each set of simulations at each equilibration temperature (300, 600, 900K), density (3.18, 3.30, and 3/42 g/cm^3) and using either the `cooling` (Fig. S1) or `quenching` (Fig. S2) regime. These fingerprints were then evaluated with respect to the experimentally determined ratios of Al(IV:V:VI) as obtained from the experimental 1D NMR spectrum shown in Fig. 2A. Simulations with matching fingerprints to experiment were selected to construct the amorphous model. The two fingerprints within error of the experimental Al coordination environments are shown in Fig. S2 which are the 3.18 g/cm^3 300K and 600K fingerprints. The main text shows results from the 300K model, and the supplementary text shows the corresponding results from the 600K model (see Fig. S5, Fig. S12, and Fig. S13). In addition, we have shown the radial distribution functions (RDF) for each model from Fig. S1 and Fig. S2 in comparison with the 1997 Lamparter *et al* RDF for an anodic film of alumina in Fig. S3. This RDF from 1997 is another tool for evaluating the model's similarity to experimental amorphous alumina, however it is important to note that the RDF is obtained using a different preparation method, temperature, and set of precursors and so is unlikely to be a perfect match to the experiments examined in this work. The results are shown in Fig. S4, and show some differences trends between model conditions, but overall a similar RDF for each set of input parameters, further highlighting the importance of comparing our results to coordination environments obtained from NMR experiments from ALD deposited alumina.

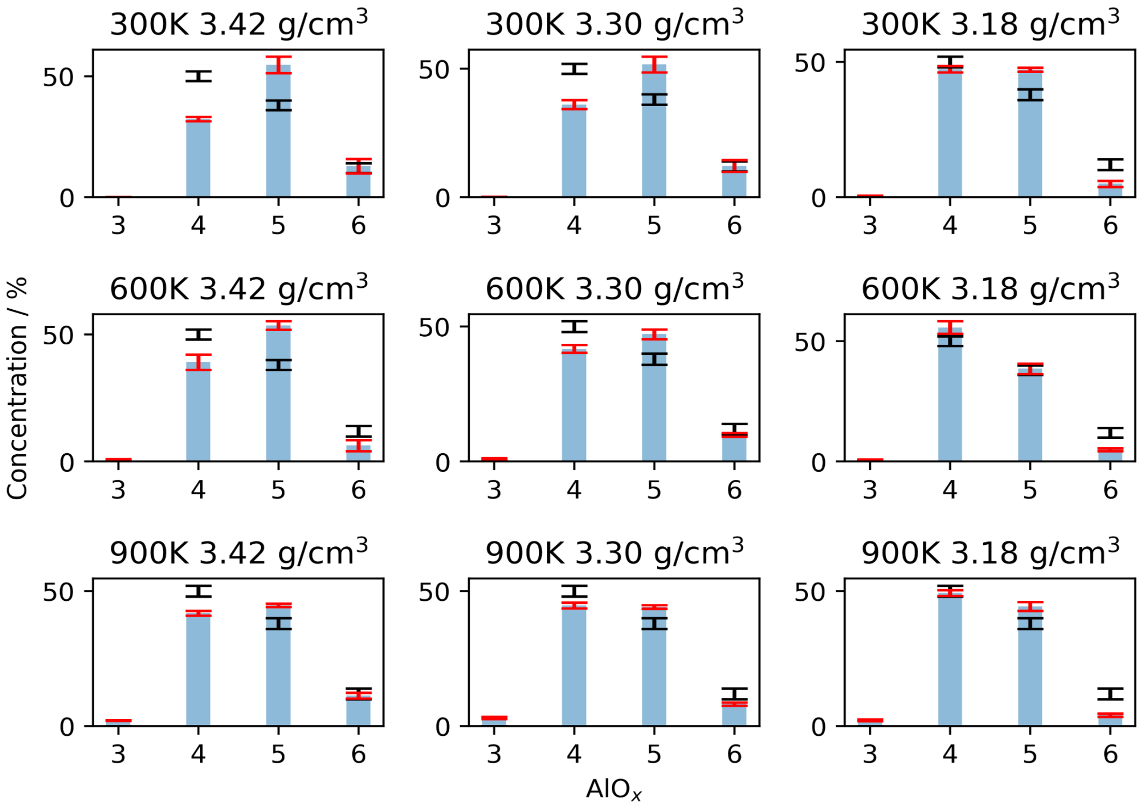

Figure S 1: *Fingerprints for 'quench' method.* Percent coordination averaged across AIMD runs for all simulations using the 'quench' method (see Methods, Structure Generation). Black bars show the error bar for experimental coordination fractions of 50%, 38%, and 12% ± 2%. Blue bars with red error bars show the average concentration of each coordination environment $AlO_{3,4,5,6}$ present across the 3 simulations run with those initial conditions. Of the 9 initial conditions shown here, none were within error of the experiment.

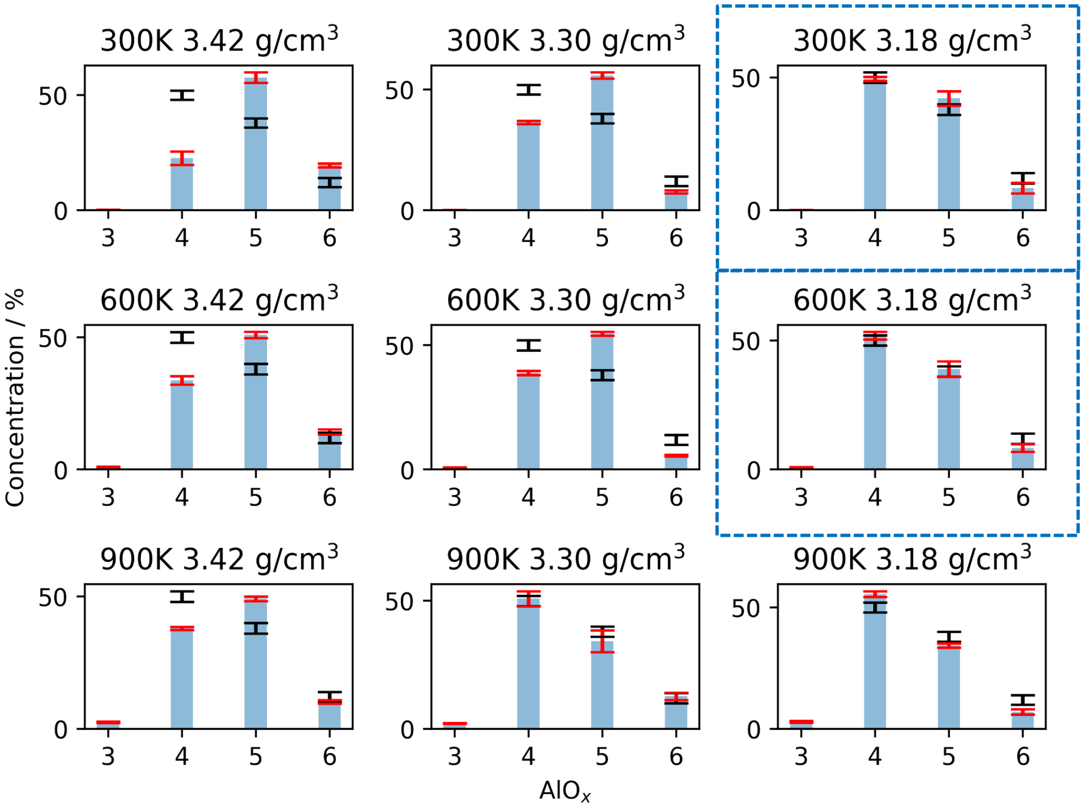

Figure S 2: *Fingerprints for 'cool' method.* Percent coordination averaged across AIMD runs for all simulations using the 'cool' method. Black bars show the error bar for experimental coordination fractions of 50%, 38%, and 12% ± 2%. Blue bars with red error bars show the average concentration of each coordination environment $AlO_{3,4,5,6}$ present across the 3 simulations run with those initial conditions. Of the 9 initial conditions shown here, two were within error of experiment, and are highlighted by the blue dashed boxes surrounding the histograms. The 300K equilibration temperature with a density of 3.18 g/cm^3 and the 600K equilibration temperature with a density of 3.18 g/cm^3. The main text shows results from the 300K model, and the supplementary text shows the corresponding results from the 600K model (see Fig. S5, Fig. S12, and Fig. S13).

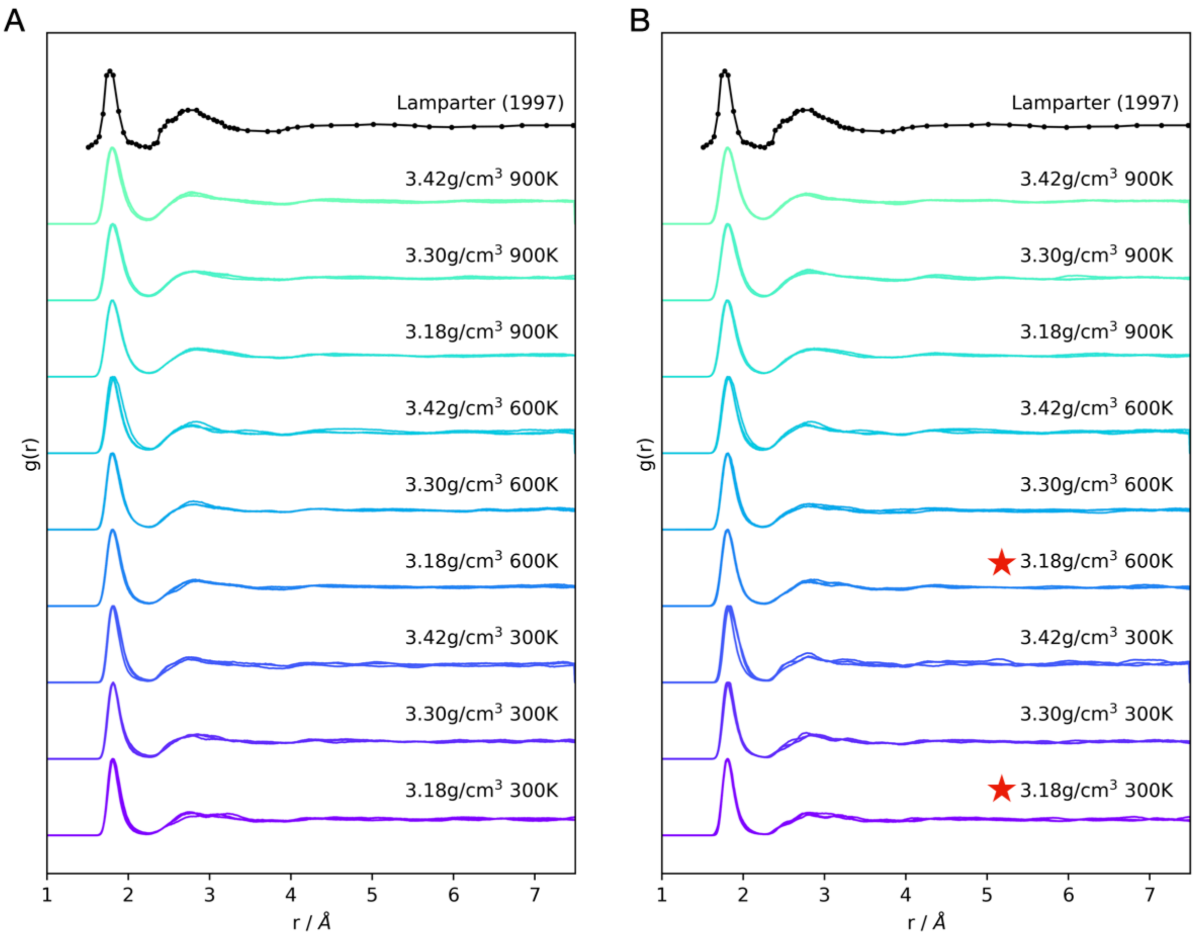

Figure S3: RDFs for all 54 simulations run using either the quench method (A) or cooling method (B) as described in the methods section, compared to the RDF from Lamparter *et al* on an anodic film of alumina [1]. These results show that all models had a similar first peak describing the Al-O bond length between 1.7 and 1.9 Å with slight differences in the broadness of the second peak between 2.4 and 3.5 Å. The two red starred RDFs correspond to the two models which fit the fingerprinting criteria. The experimental data is obtained from Lamparter and Kneip, 'Structure of Amorphous Al2O3' *Physica B: Condensed Matter* (1997) [1]with permission from Eselvier.

2. NMR Data (Experimental and Computational)

2a. Computation of NMR spectra

In order to align the calculated NMR spectra with experimental results, we used a set of experimentally known NMR reference compounds in order to reference the ^{27}Al isotropic shieldings. As shown in Table S1, a set of 8 Al containing compounds were optimized in DFT, and their NMR parameters were calculated and compared to the experimental reference parameters. Details of the parameters such as plane wave cutoff, k-point spacing, and convergence criteria for the DFT calculated NMR spectra are given in the Methods. From the reference and DFT calculated shieldings in Table S1, we then plotted their values in Fig. S4, according to the relation $\delta_{iso} = \sigma_{ref} - m\sigma_{iso}$. This plot yielded values of $\sigma_{ref} = 635.4$ ppm and $m = 1.16$, which were then used to reference the NMR spectra in Fig. 2C and D. Following this referencing, it is now possible to compare the calculated NMR spectra to the experimental NMR spectra, as we have done throughout the main text, and as we will show in more detail in the following sections.

Table S 1: Comparison of the ^{27}Al NMR parameters obtained by DFT and experiment. These isotropic shielding values were used for the linear fitting in Fig. S4. The choice of reference structures for the ^{27}Al NMR was based on the fitting used to calculate ^{27}Al NMR shieldings in Mizoguchi et al[2].

Chemical Formula	Space Group	δ_{iso} Exp. (ppm)	σ_{iso} DFT (ppm)	C_Q Exp. (MHz)	C_Q DFT (MHz)	Reference
Al_4C_3	$R\bar{3}m$	111.2	459.4	15.58	14.1	[3]
		120.1	443.7	15.83	17.1	[3]
AlF_3	$R\bar{3}H$	-14.7	565.3	-	0.1	[4]
		-16.0	566.0	-	0.3	[4]
$LaAlO_3$	$Pm\bar{3}m$	0	547.3	0.2	0.0	[5]
$LaAlO_3$	$R\bar{3}c$	0.03	547.1	-	0.1	[5]
$LiAlO_2$	$P4_12_12$	17	535.3	3.2	3.0	[6]
$LiAlO_2$	$I4_1/amd$	82.0	476.7	2.8	3.6	[6]

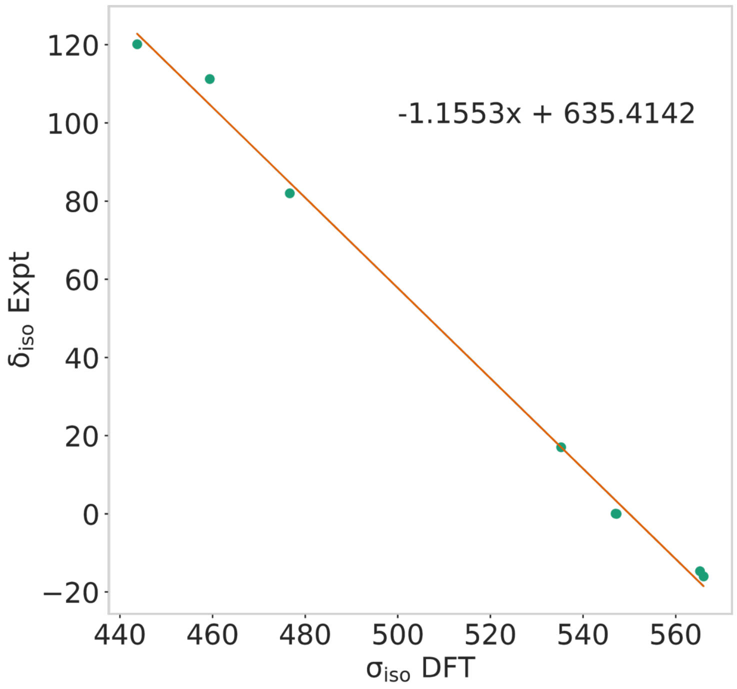

Figure S 4: GIPAW NMR calculated shieldings for a selection of Al-containing structures from the ICSD, listed in Table S2. The orange line is a linear fit, from which we can extract the values of m and σ_{ref} according to the relation $\delta_{iso} = \sigma_{ref} - m\sigma_{iso}$. These values are $\sigma_{ref} =\ 635.4$ ppm and $m = 1.16$, and are used to reference the NMR spectra in Fig. 2C and D.

2b. Experimental NMR spectra and comparison with DFT results

Herein we provide details of the calculated shifts, shieldings, and quadrupolar coupling constants both calculated and experimentally determined for the amorphous alumina model and experimental substrate, respectively. Table S2 shows a summary of the DFT computed isotropic shieldings to the NMR parameters obtained from fitting the experimental spectrum collected at 16.4 T. This experimental 1D spectrum was used for identifying the coordination environments used for the fingerprinting and is shown in Fig. 2A.

Table S 2: NMR parameters for DFT GIPAW NMR compared to experimental parameters for the 300K simulation. The computed δ_{iso} value is calculated as the lineshape maximum of each Al environment Al(IV:V:VI) from the DFT calculated 1D spectrum of isotropic shifts shown in Fig. 2C. The C_Q values from DFT represent the average over the values for each Al environment Al(IV:V:VI) across the total 2160 sites. Experimental values are extracted using DMFit[7] and the Czjzek model (GIM case) commonly applied to amorphous quadrupolar nuclei such as ^{27}Al[8]. The fit yields average δ_{iso} chemical shift and the chemical shift distribution (ΔCS) and the average magnitude of C_Q, $\langle|C_Q|\rangle$.

**Coordination**	**Fraction**	δ_{iso} **(ppm)**	$\langle\|C_Q\|\rangle$ **(MHz)**	σ **(MHz)**
**DFT GIPAW NMR**				
**Al(IV)**	49	74	12.9	4.1
**Al(V)**	42	45	10.5	3.9
**Al(VI)**	8	10	9.1	3.4
**1D Czjzek Fitting (*B*₀ = 16.44 T)**				$\sigma = \langle\|C_Q\|\rangle/1.995$ **(MHz)**
**Al(IV)**	50	74±14*	10.9	5.4
**Al(V)**	38	41±13*	7.8	3.7
**Al(VI)**	12	11±10*	7.9	3.8

*These values correspond to δ_{iso} ± ΔCS

Spectra were fitted using DMFit[7] with the implemented Czjzek model (Gaussian isotropic model) [9–11] The fit yields the averaged isotropic chemical shift (δ_{iso}), the chemical shift distribution (ΔCS), and the mean quadrupolar coupling constant (C_Q), reflecting the distribution in values of C_Q [12]. Due to the large number of parameters to be optimized, we followed the procedure by Baggetto *et al.* [13] and first fitted the 3QMAS to extract the chemical shift distribution (ΔCS) of each Al coordination environment. The quantitative 1D spectra (more on why MQMAS was not sufficient below in section 3c) were then fitted while keeping the previously obtained value of ΔCS fixed. ^{27}Al 1D NMR spectra were measured on the same 1000 ALD layer on silicon sample at three different magnetic fields strengths (Fig. S5 and Fig. 3) to explore the validity of the fits, the contribution from the second-order quadrupolar broadening decreasing as the strength increases. The collection of NMR parameters including those obtained from fits at different

magnetic fields is given in Table S 2 and Table S 3. The experimental errors given in Table S3 correspond to estimates obtained by visually evaluating the goodness of fit by changing the parameter and re-optimizing the fit. The many different variables make the optimization challenging, however changes to the parameters in the range given by the errors still resulted in a reasonable fit. Of note, in general the values of $\langle|C_Q|\rangle$ increased as the field was increased, indicating that the lower field spectra either fail to excite all the signals from the sites with large values of C_Q or that they are not appropriately modelled in the fits.

Table S 3: Comparison between ^{27}Al NMR parameters obtained from DFT and experiments performed at three different fields with fits shown in Fig. S6. Throughout the manuscript, the experimental fraction for each coordination environment is taken from the fits to the 700 MHz, 16.44 T spectrum. This spectrum provides higher resolution than the 500 MHz, 11.75 T spectrum and longer measurement times and a larger rotor was used, in comparison to the conditions used to collect the 1 GHz spectra, improving the signal to noise ratio and the confidence in the fitted values.

**Al Coordination**	***B*$_0$**			
	**11.75 T**	**16.44 T**	**23.49 T**	**DFT**
Average δ_{iso} (±2) / ppm				
Al(IV)	70	74	75	74
Al(V)	39	41	44	45
Al(VI)	10	11	20	10
ΔCS(±2) / ppm				
Al(IV)	14	14	14	-
Al(V)	13	13	13	-
Al(VI)	10	10	10	-
$\langle|C_Q|\rangle$ (±0.5) / MHz				
Al(IV)	9.8	10.9	16.0	12.9
Al(V)	7.6	7.8	10.8	10.5
Al(VI)	6.0	7.9	9.2	9.1
Fraction (±2) / %				
Al(IV)	51	50	56	49
Al(V)	41	38	34	42
Al(VI)	8	12	10	8

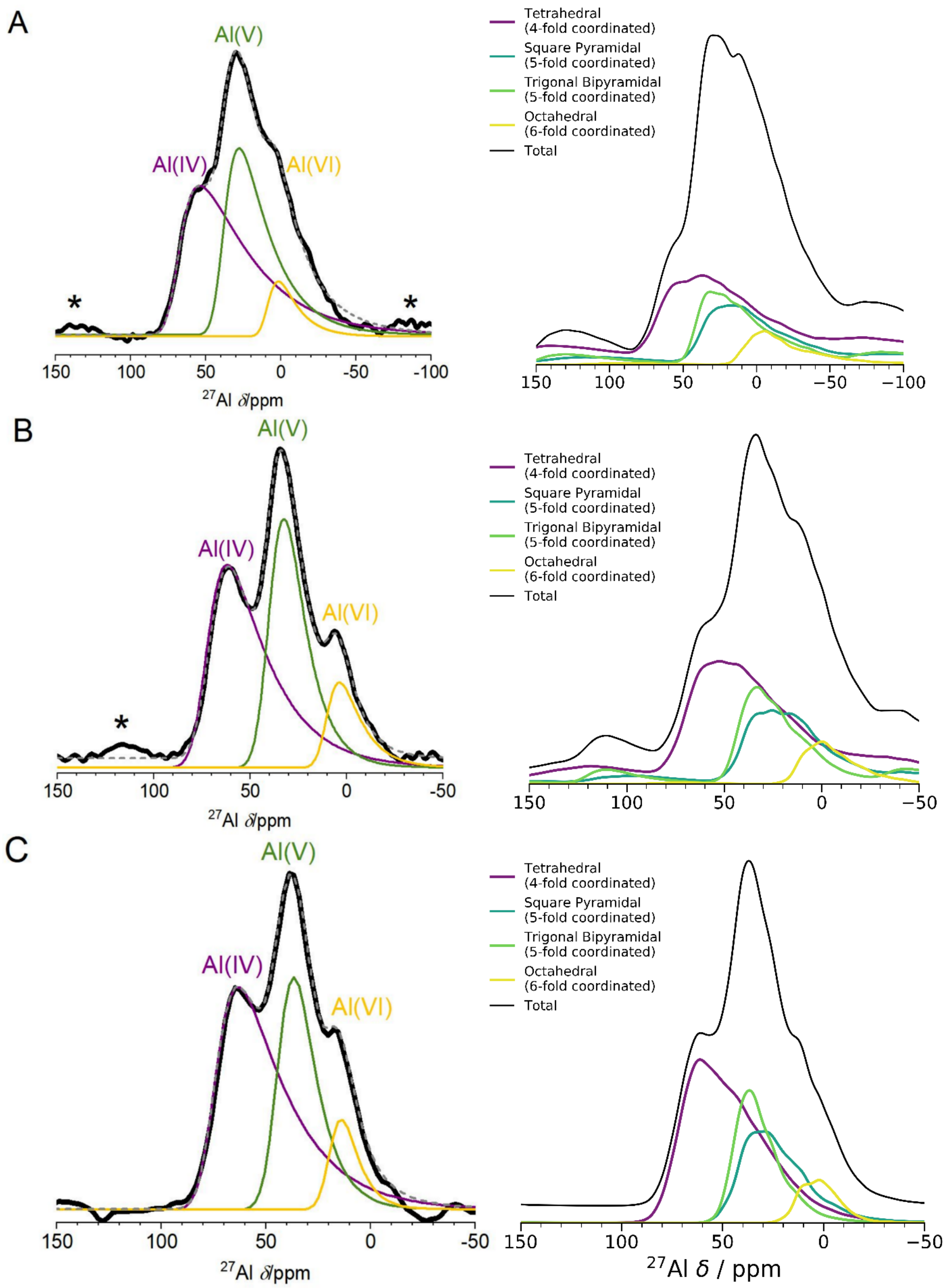

Figure S 5: 1D NMR spectra for the 600K, 3.18 g/cm³ cooling initial conditions, including quadrupolar lineshapes calculated using SIMPSON[14] at three different fields (A 11.5 T, B 16.4 T, and C 23.5 T) corresponding to the fields used in the experimental NMR (left). In each case, as the field increases, the sharpness of the three NMR peaks increases, as is expected. Additionally, the 23.5 T field was calculated with a 40 kHz MAS speed while both 11.5 T and 16.4 T were calculated with a 14 kHz spinning speed, as used in the experiments. Each of these peaks is comprised of two types of lines shapes, large C_Q, broad line shapes with low intensity and small C_Q, sharp line shapes with high intensity. Given the lack of separation between the peaks, even at 23.5 T, this suggests that the large C_Q Al sites smear out some of the sharper peaks. Spinning sidebands are marked with an asterisk.

2c. Details on choosing a spectrum for fingerprinting and MQMAS results

In an ideal case, an MQMAS spectrum would have been the most suitable to guide the structure matching, supported by 1D spectra to extract more accurate values of the relative intensities of the peaks corresponding to the different environments: MQMAS spectra are not quantitative when sites with very different C_Qs are excited, and so common practice is to use the parameters obtained from fitting the MQMAS spectrum to fit the 1D spectrum keeping all parameters apart from the intensity fixed. This would be the ideal starting point, but for the ^{27}Al 1D spectrum at the 16.44 T field, this method still did not give a satisfactory fit. The nature of the fit suggests that the environments with too large C_Q's were MQMAS "invisible" – or not detected, since they could not be efficiently excited with the rf pulse strengths used. This is due to the inefficient excitation of the triple quantum transition in the case of a large C_Q (largely because the rf excitation bandwidth of the triple quantum transition is smaller than the second-order broadening). This led to a lower average C_Q extracted from the MQMAS spectrum, an upper C_Q limit of around 6-7MHz being measured in these experiments. To fit the 1D spectrum (16.44T) satisfactorily, peaks with a higher C_Q were required. Fig. S6 shows that additional 4 and 5-coordinate Al environments (Al(IV) and Al(V)) with the same chemical shift but a higher C_Q (10-12MHz) are required to achieve a satisfactory fit for the 1D spectrum based on the MQMAS parameters. This 5-component fit is less physically meaningful and was included to demonstrate that MQMAS cannot be relied on for intensity and C_Q values for this system. In addition to these large C_Q components being likely to exist experimentally, we also see a range of C_Q values in the DFT calculated NMR spectrum ranging from 5 to 25 MHz as shown in Fig. S7, matching the experimental evidence for large C_Q sites.

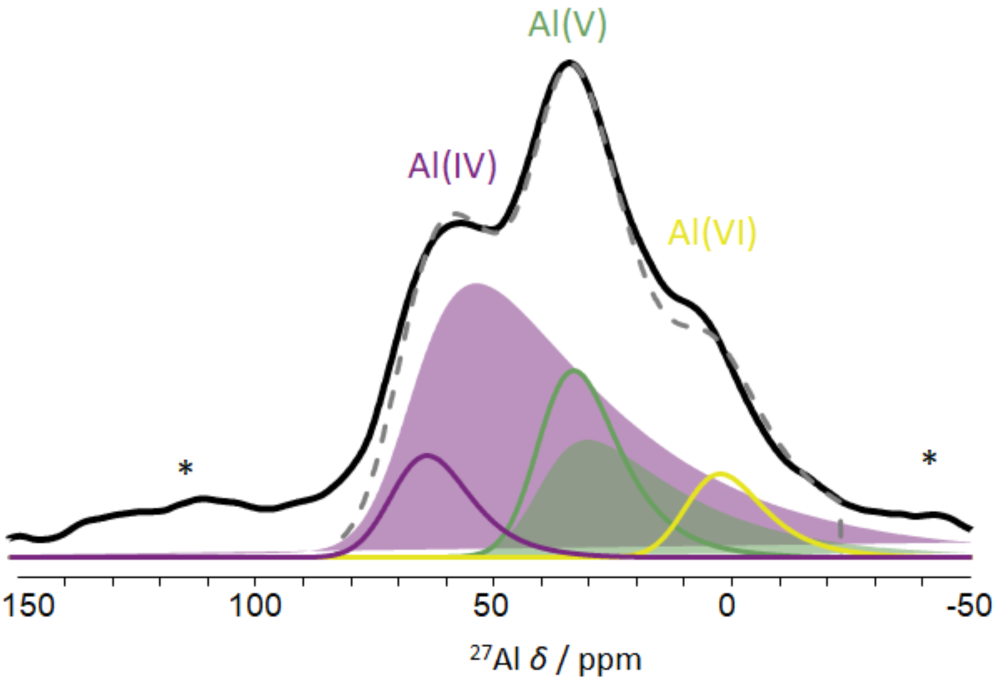

Figure S 6: Experimental ^{27}Al 1D NMR spectrum collected at 16.44 T (black), fitted using a 5 component fit in which 3 components are taken from the fit of the MQMAS spectrum (lines) and 2 additional components with a higher C_Q corresponding to MQMAS "invisible" (filled areas) environments. The MQMAS components were fixed and only the intensity was optimized while the additional components were fixed at the same average chemical shift as their MQMAS counterpart but all variables were optimized. The parameters obtained from the overall fit for each component are given in Table S4. Spinning sidebands are marked with an asterisk.

Table S 4: ^{27}Al NMR parameters obtained if a 5-component fit according to Fig. S6 is considered while keeping the chemical shifts constant and fixing some of the C_Q values (of MQMAS peaks).

**Al Coordination**	**Average δ_{iso}/ppm**	**Average C_Q / MHz**	**Ratio / %**
**Al(IV)**	69±1	6.6 (MQMAS)	8
		12.0 (additional)	54
**Al(V)**	40±1	6.4 (MQMAS)	15
		10.0 (additional)	16
**Al(VI)**	9±1	6.8 (MQMAS)	7

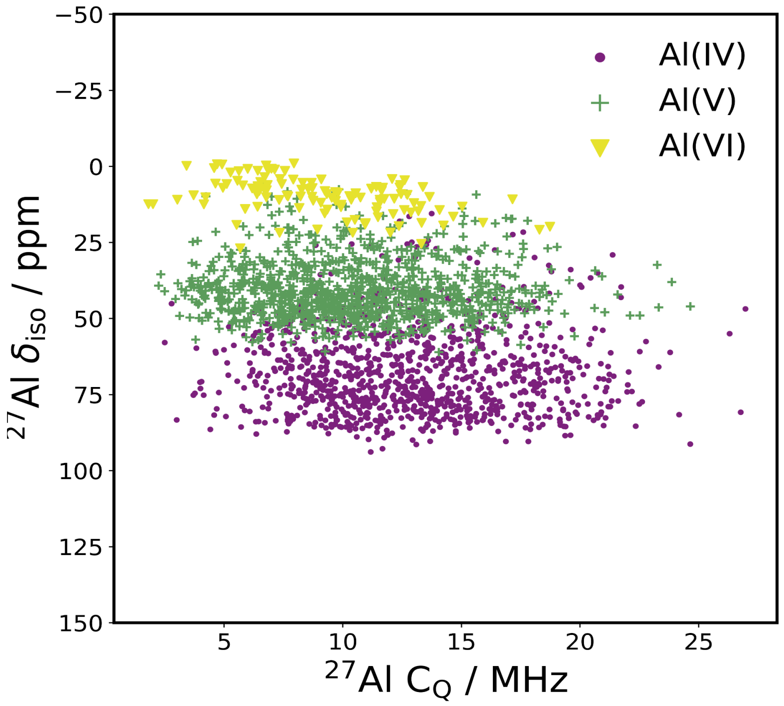

Figure S 7: Plot of the GIPAW NMR calculated C_Q vs. the calculated δ_{iso} values for the 300K, 3.18 g/cm^3, cooling initial conditions. This plot shows that there is a large range of C_Q values for both Al(IV) of more than 20 MHz, but Al(VI) C_Q varies across only 15 MHz. This indicates that quadrupolar effects will play a large role in the overall experimental lineshape of the NMR spectrum, and also suggests that there may be large C_Q environments in the experimental spectrum as well.

2d. Experimental NMR Referencing and Background

All ^{27}Al experiments were performed with ZrO_2 rotors and plastic caps, so that the Al background from the container was excluded. The stator of the NMR probe is sometimes made of macor (alumina) due to its heat resistance properties useful for high temperature measurements. This was the case for the probehead used at the 16.44 T field. While the signal of the solid surrounding is generally negligible under MAS conditions, however, considering the small sample amount of the ALD thin film, this assumption was tested. Spectra were acquired with the same acquisition parameters on both the filled and empty rotors. As can be seen in Fig. S8 the background contribution is small, and has thus been ignored in any fits to the spectra. Finally, ^{27}Al 1D spectra were measured on γ-Al_2O_3 at B_0 = 16.44 T, and one is shown for comparison in Fig. S9.

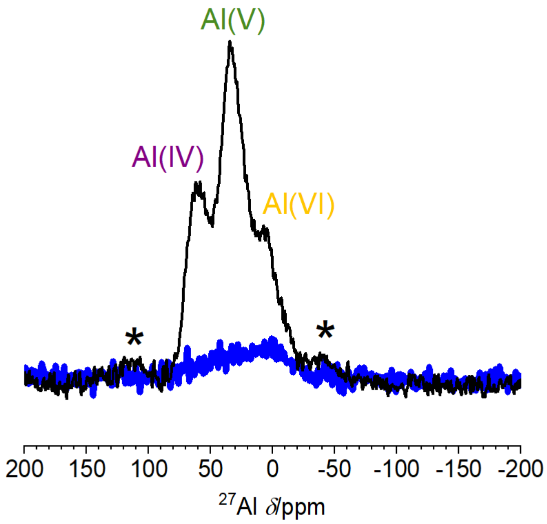

Figure S 8: ^{27}Al spectra at 16.44 T showing the spectrum with sample (black) and without sample (blue), the latter being performed to rule out any significant background contribution to the spectra; measurements were performed using the exact same parameters, i.e. pulse sequence, receiver gain, number of scans and delay between pulses.

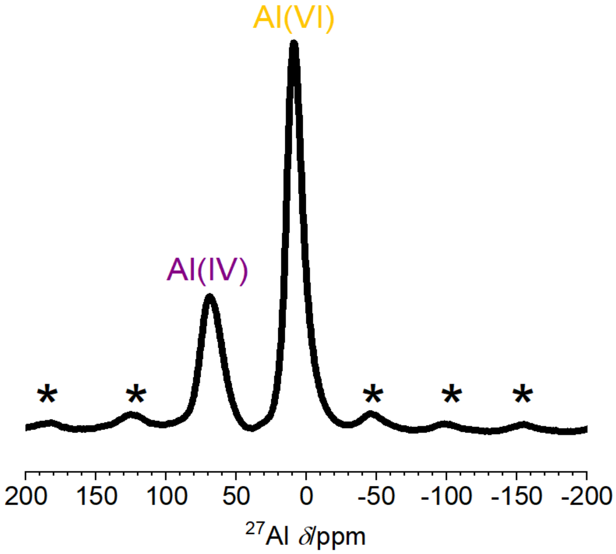

Figure S 9: A 1D ^{27}Al spectrum of γ-Al_2O_3 was included to show the reference ^{27}Al NMR isotropic shifts of Al(IV) and Al(VI). These shifts are 10 ppm for Al(VI) and 66 ppm for Al(IV).

3. Calculated XAS spectra

As detailed in the Methods section on *DFT-Calculated Spectroscopy*, we have used a core-hole method to calculate the absorption spectra for the structures presented in this work. Fig. S10 illustrates the importance of using this method in order to obtain the experimentally relevant absorption peaks in the XAS spectra by comparing two XAS spectra for α-Al_2O_3. The non core hole spectrum has a maximum peak at 1573 eV, where the secondary peak in the experimental spectrum lies, and fails to reproduce the location of the main absorption edge at 1567 eV. For this reason, we chose to use the core hole method as described, in order to produce spectra which were consistent with experiment.

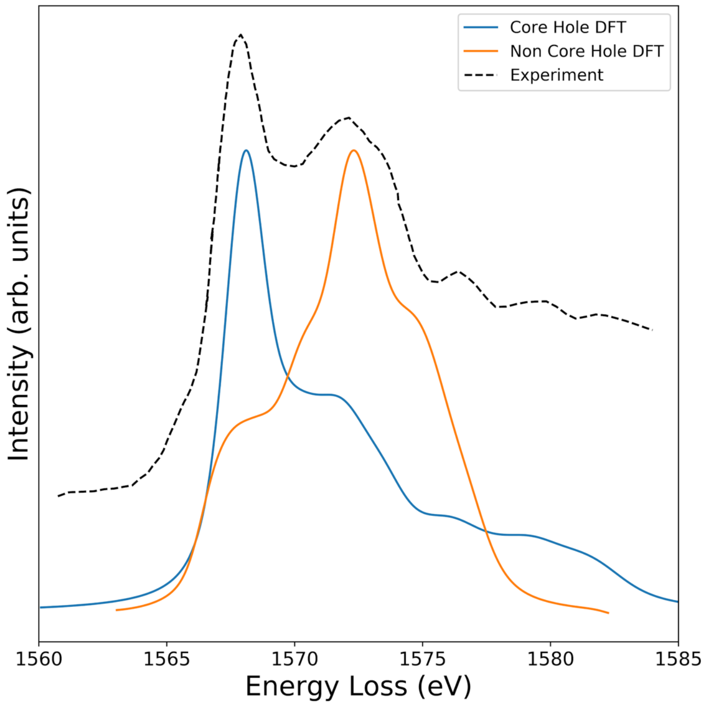

Figure S 10: DFT calculated XAS of α-Al_2O_3 with applied correction from Mizoguchi *et al.*[2] and shifted 10.1 eV to match the experimental first peak from Cabaret *et al*[15]. The peak positions in both DFT and experiment are within less than 1 eV, and only the intensity of the peaks varies. In general, the DFT intensities are lower than experiment, as a result of a limited number of plane-waves used to calculate the states at higher Energy Loss values. The orange line shows the results of the XAS calculation without including a core-hole on the Al site, which has a completely different lineshape to the experiment, indicating that it is necessary to include core-holes in the XAS calculations.

4. Effect of distortions on the electronic states of alumina

In the XAS spectra shown in Fig. 5, the pre-edge peak was reproduced in the model by contributions from several core holes placed on Al(IV) and Al(V) environments. In order to explore the nature of this pre-edge peak, we calculated an eDOS for two systems, one with a pre-edge peak and one without. The main difference between these two sites was their level of distortion, as measured by the crystal symmetry metric (CSM). For the core-hole placed on a distorted site, a state in the band gap at 1.5 eV arises (Fig. S11 bottom) whereas for the symmetric site no state exists in the band gap (Fig. S11 top). This suggests that both Al(IV) and Al(V) sites are more prone to distortion and thus give rise to states in the band gap of this insulating phase, which lead to the pre-edge shown in the XAS spectrum. This pre-edge in the amorphous case is likely a result of an Al-O bond lengthening resulting in a transition from *1s* to a mixed *s,p* state; in the distorted phase Mullikan charge analysis shows that 3 of the 4 Al-O bonds in the tetrahedra have a population between 0.12 and 0.16, while one (with bond length 1.98 Å) has a population of 0.07. In the symmetric case, all four Al-O bonds have populations between 0.12 and 0.16, indicating that all four of these bonds are populated, thus no pre-peak is observed. In most cases in the literature, the pre-edge from Al(IV) is not observed[16], and thus is unique to ALD deposited a-Al_2O_3.

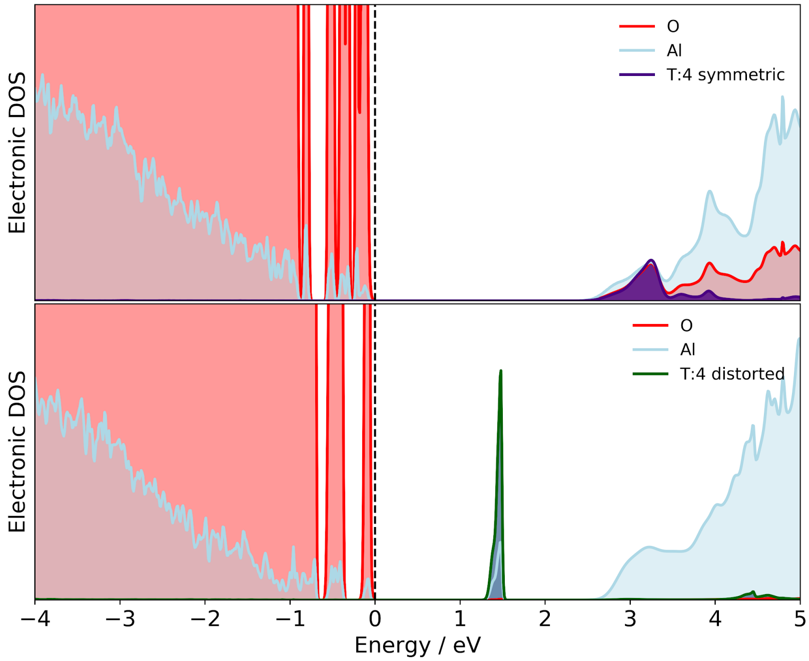

Figure S 11: The electronic density of states is calculated with a core-holes placed on a tetrahedral symmetric site (CSM < 2.5) and a distorted site (CSM > 2.5). The distorted site has a state in the bandgap while the symmetric site does not, suggesting that the pre-peak arising in the XAS spectra arises from transitions within the distorted coordination environment surrounding the core-hole atom. The distorted site (CSM 3.6) contains mixed states at 1.5 eV above the valence band. In the pristine tetrahedral case (CSM 0.7), this state at 1.5 eV is not present, and the density of states suggests that transitions start to occur at around 2.75 eV above the Fermi level.

5. Additional spectra for amorphous alumina model at 600K 3.18 g/cm^3

As shown in the fingerprinting step of our approach to generating an amorphous model, two fingerprints were within error of the experimental values. These were fingerprints from the cooling regime, at a density of 3.18 g/cm^3 at 600K and 300K. The 300K results are shown in the main text of the paper, and the 600K results are shown here in the Supplemental information. The NMR spectra, at three fields are shown in Fig. S5 and follow the same trends of increasing resolution of the Al(IV:V:VI) peaks at higher field strength, as well as the same Czjzek-like shape of the coordination environment separated lineshapes, indicating that both this model and the one presented in the paper are representative of the atomic level structure of alumina. As shown in Fig. S12 this model also reproduces the a, b, and c peaks from the experimental XAS spectra, although the peak at c is less well defined than in the model at 300K shown in the main text (Fig. 5) but the peak positions are reproduced well in the model. Finally, the electronic density of states shown in Fig. S13, shows a localized set of states with Al s character at the conduction band minimum, as well as a band gap of 2.6 eV, which is consistent with the band gap identified in the 300K model. These results show that our fingerprinting method was robust enough that two fingerprints which match the criteria we have set (i.e. being within the experimental error of the ratio of Al(IV:V:VI) environments) all produce models of amorphous alumina which have structural and electronic properties of experimental amorphous alumina. This underlines the reproducibility of our method.

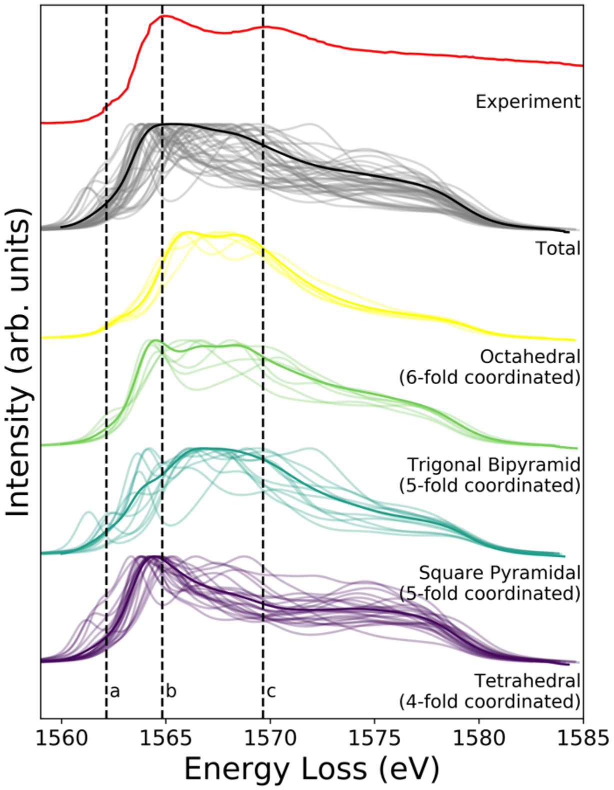

Figure S 12: XAS spectra obtained from the 600K, 3.18 g/cm^3, cooling fingerprint compared to the experimental (red) XAS from the ALD deposited Al_2O_3 sample. The grey lines show each individual core-hole spectra calculated at a single Al site in the configuration from AIMD, and the solid black line is the sum of those spectra. The colored spectra show each geometric environment's core-hole spectra; thin lines are individual core-hole spectra, thick lines are the sum of each geometric site's spectra. This amorphous model has the same features at peaks a, and b as in experiment, indicating that the electronic states in this model agree with experimental electronic states. Peak c has a lower intensity, suggesting the octahedral environments in this model are less pronounced.

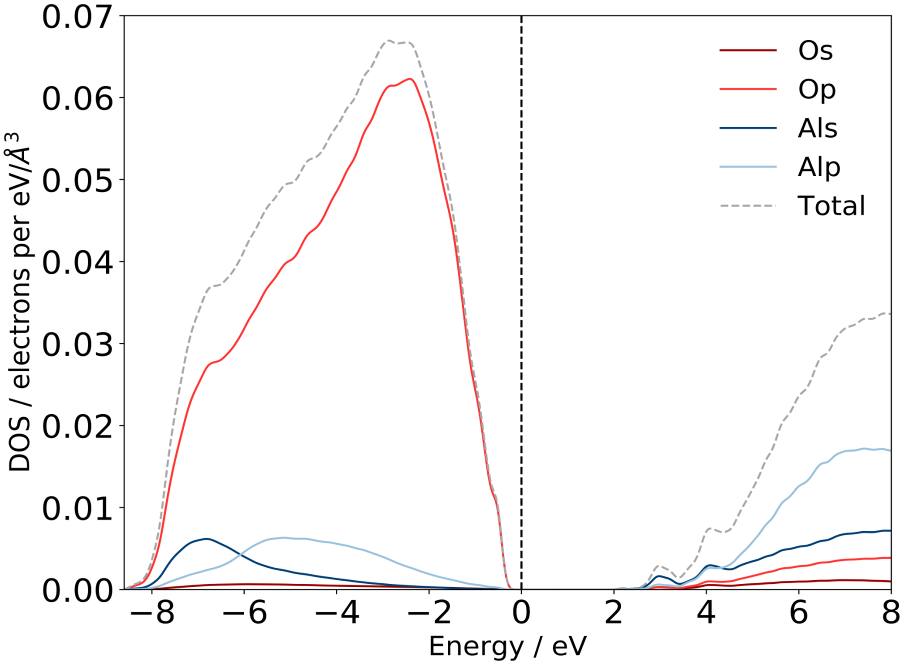

Figure S 13: The electronic Density of States (eDOS) for the 600K, 3.18 g/cm^3, 'quench', model of a-Al_2O_3. Total electronic density of states separated by atom and orbital contribution shows that a-Al_2O_3 is a wide bandgap insulator, with a gap of 2.6 eV, which is comparable to the 2.6 eV band gap from the 300K model. There are equivalent localized states at 3.2 and 4.2 eV above the Fermi level which have Al *s* character and mixed Al *s,p* character respectively, as in the 300K model (Fig. 6).

6. Assessing stability of the method

In the previous section we showed results from a fingerprint which also had coordination fractions within error of experiment. To illustrate that the fingerprints with coordination fractions that did not lie within experimental error have distinct NMR spectral signatures from those shown in the main text, we have repeated the spectroscopic analysis for a model with configurations generated from the quenching regime at a final temperature of 600 K with a density of 3.30 g/cm^3. This fingerprint contains Al(IV/V/VI) environments in fractions of 41%, 50%, and 8% compared to experimental fractions of 50%, 38%, and 12% ± 2%, and therefore was not used to create an amorphous model in the main text, but is presented here to show how the results of a model with a fingerprint that did not match experiment, compare. In the NMR spectra shown in Fig. S14 it is clear that there are an excess of Al(V) environments in the model, and the Al(V) peak intensity is increased compared to that for Al(IV/VI). Furthermore, especially in the modelled spectrum at 23.3T in Fig. S14C we see that the Al(IV) environments, which are experimentally known to have the highest quadrupolar nature, have a gaussian shaped spectrum, suggesting that the geometries of these environments are also not comparable to experiment.

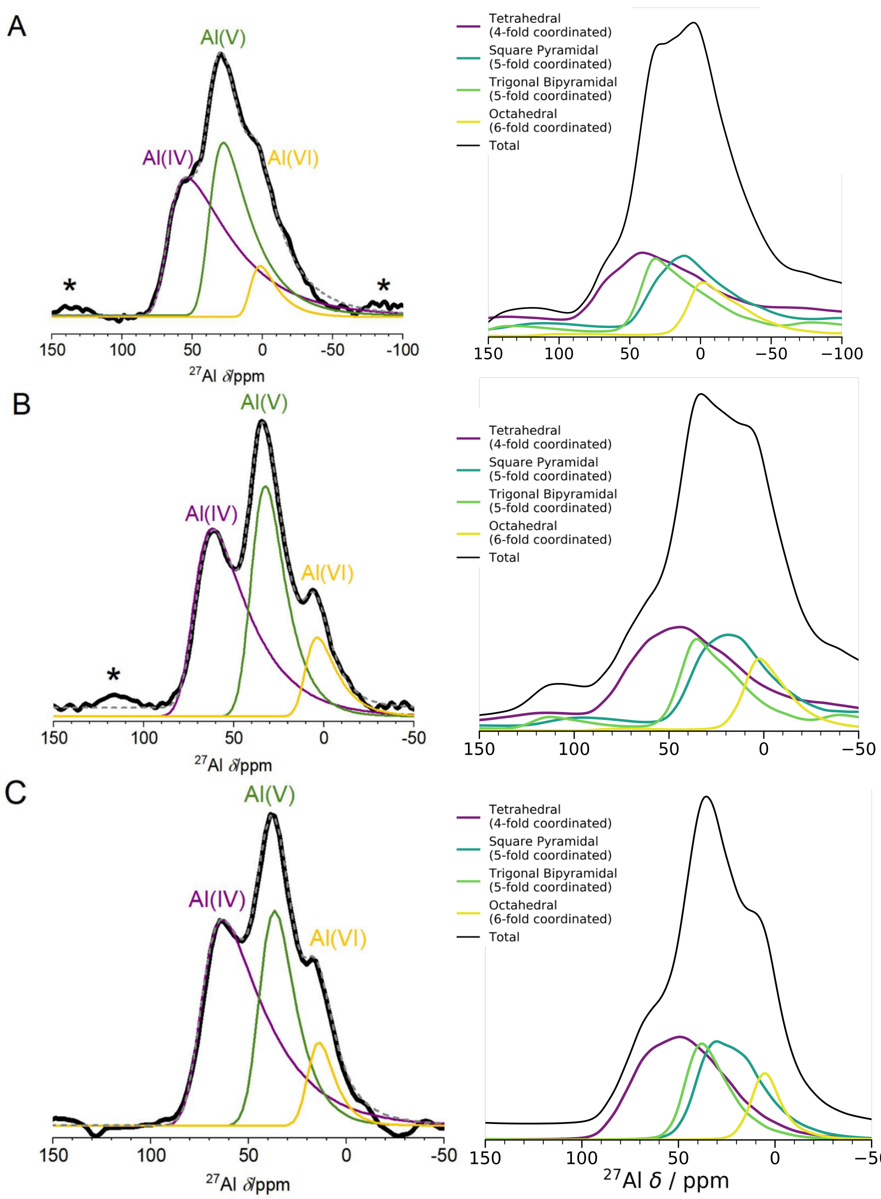

Figure S 14: (left) Experimental 1D NMR spectra. (right) 1D NMR spectra for the 600K, 3.30 g/cm^3 quench initial conditions, including quadrupolar line shapes calculated using SIMPSON[14] at three different fields (A 11.5 T, B 16.4 T, and C 23.5 T) corresponding to the fields used in the experimental NMR (left). This fingerprint corresponds to Al(IV/V/VI) environments in fractions of 41%, 50%, and 8% compared to experimental fractions of 50%, 38%, and 12% ± 2%. The larger fraction of Al(V) environments is clearly visible in the height of the Al(V) peak in all 3 fields in the right panel of simulated NMR spectra. These chemical shifts are not in line with experiment, as expected given the difference in coordination environments.

7. Supplementary References

1 P. Lamparter and R. Kniep, *Physica B Condens Matter*, 1997, **234–236**, 405–406.
2 T. Mizoguchi, I. Tanaka, S.-P. Gao and C. J. Pickard, *Journal of Physics: Condensed Matter*, 2009, **21**, 104204.
3 T. Bräuniger, C. V. Chandran, U. Wedig and M. Jansen, *Z Anorg Allg Chem*, 2011, **637**, 530–535.
4 J. C. C. Chan and H. Eckert, *J Non Cryst Solids*, 2001, **284**, 16–21.
5 F. Blanc, D. Middlemiss, L. Buannic, J. Palumbo, I. Farnan and C. Grey, *Solid State Nucl Magn Reson*, 2012, **42**, 87–97.
6 D. Müller, W. Gessner and G. Scheler, *Polyhedron*, 1983, **2**, 1195–1198.
7 D. Massiot, F. Fayon, M. Capron, I. King, S. St´, S. le Calvé, C. Calvé, B. Alonso, J.-O. Durand, B. Bujoli, Z. Gan and G. Hoatson, *Magn. Reson. Chem*, 2002, **40**, 70–76.
8 G. Engelhardt and H. Koller, *Magnetic Resonance in Chemistry*, 1991, **29**, 941–945.
9 G. le Caër and R. A. Brand, *Journal of Physics: Condensed Matter*, 1998, **10**, 10715.
10 G. le Caër, B. Bureau and D. Massiot, *Journal of Physics: Condensed Matter*, 2010, **22**, 065402.
11 G. Czjzek, J. Fink, F. Götz, H. Schmidt, J. M. D. Coey, J.-P. Rebouillat and A. Liénard, *Phys Rev B*, 1981, **23**, 2513.
12 U. Werner-Zwanziger, A. L. Paterson and J. W. Zwanziger, *J Non Cryst Solids*, 2020, **550**, 120383.
13 L. Baggetto, V. Sarou-Kanian, P. Florian, A. N. Gleizes, D. Massiot and C. Vahlas, *Physical Chemistry Chemical Physics*, 2017, **19**, 8101–8110.
14 M. Bak, Rasmussen. JT and N. Nielsen, *J Magn Reson*, 2000, **147**, 296–330.
15 D. Cabaret, P. Sainctavit, P. Ildefonse and A.-M. Flank, *Journal of Physics: Condensed Matter*, 1996, **8**, 3691.
16 J. A. van Bokhoven, A. M. J. van der Eerden and D. C. Koningsberger, *J Am Chem Soc*, 2003, **125**, 7435–7442.
